# Different Elemental Compositions and Potential Functions of Vacuoles in *Bolivina spissa* (Foraminifera, Rhizaria) Based on Cryo‐SEM‐EDS Analyses

**DOI:** 10.1111/jeu.70044

**Published:** 2025-08-31

**Authors:** Julien Richirt, Satoshi Okada, Yoshiyuki Ishitani, Nicolaas Glock, Katsuyuki Uematsu, Hidetaka Nomaki

**Affiliations:** ^1^ SUGAR, X‐Star Japan Agency for Marine‐Earth Science and Technology (JAMSTEC) Yokosuka Japan; ^2^ Institute for Geology University of Hamburg Hamburg Germany; ^3^ Marine Works Japan Ltd. Yokosuka Kanagawa Japan

**Keywords:** anoxic metabolism, benthic foraminifera, cryo‐SEM‐EDS techniques, denitrification, N and P intracellular storage, P‐based metabolism, vacuolar system

## Abstract

Benthic Foraminifera exhibit diverse adaptations to low oxygen (O_2_) environments, including denitrification, a rare trait among eukaryotes. Denitrifying species store intracellular nitrate (NO_3_^−^), possibly within vacuoles, and contribute significantly to the global marine nitrogen (N) cycle. Additionally, widespread phosphate (PO_4_^3−^) accumulation suggests a role in supporting metabolism under O_2_‐depleted conditions. However, the organelles storing NO_3_^−^ and PO_4_^3−^ remain unknown, limiting the mechanistic understanding of these alternative metabolic pathways. To investigate the intracellular NO_3_^−^ and PO_4_^3−^ localization in the benthic foraminifera *Bolivina spissa*, experimental incubations under varying O_2_ and NO_3_^−^ conditions followed by cryogenic fixation and scanning electron microscopy‐energy dispersive spectroscopy (SEM‐EDS) analyses were carried out. Most vacuoles were enriched in N relative to the surrounding cytoplasm, likely representing the intracellular NO_3_^−^ reservoir. The elemental mapping also confirmed phosphorus (P) enrichment in organelles resembling acidocalcisomes, likely as PO_4_^3−^, which may serve as a readily available energy source used over NO_3_^−^ storage during the transition between aerobic and anaerobic respiration. Additionally, barium‐rich vacuoles of unknown function(s) display a unique spatial distribution. This study emphasizes the effectiveness of cryogenic techniques in elucidating metabolic processes in foraminifers and other large and/or testate unicellular organisms, particularly for studying soluble compounds that have rarely been investigated.

## 1 Introduction

The oceans have been losing dissolved oxygen since the middle of the 20th century. Driven by global warming and nutrient enrichment from human activity, oxygen depletion is one of the most important changes occurring in marine ecosystems, posing a major threat to both marine life and biogeochemical processes (Breitburg et al. 2018). Therefore, understanding how marine organisms cope with oxygen depletion is especially important to assess and predict the effects of oxygen decline on marine ecosystems. While life emerged in an anoxic world, eukaryotes are generally believed to have arisen as aerobic organisms (Altenbach et al. 2012). Alternatively, the diversity in mitochondrial metabolism suggests that eukaryotes may have emerged using anaerobic metabolism (Zimorski et al. 2019), stressing the complex and intriguing history of early eukaryotic evolution. Anoxic conditions have been recurrent throughout Earth's history, occurring multiple times in large parts of the ocean (Strauss 2006), often coinciding with mass extinction events (Wignall and Hallam 1992; Kimura and Watanabe 2001). Therefore, eukaryotic microbes have been exposed to various oxygen concentrations at a geological scale, resulting in the development of various morphological, behavioral, and/or metabolic adaptations to oxygen‐depleted environments (Altenbach et al. 2012; Spicer 2016). For instance, very few unicellular eukaryotes are known to perform denitrification and respire nitrate (NO_3_^−^) instead of oxygen (O_2_), including representatives of the ciliate genus *Loxodes* (Finlay et al. 1983) and two fungi species (Usuda et al. 1995).

Benthic Foraminifera (Rhizaria) are unicellular eukaryotes distributed worldwide (Murray 2006), with some of them having a shell (test). Fossil records date their emergence back to the early Cambrian (545 Ma, (Culver 1991)), and molecular clock estimates suggest an even earlier origin during the Neoproterozoic (770 Ma, (Groussin et al. 2011)). Their ecological success may be partly attributed to their ability to cope with oxygen depletion or even absence, which is extensively studied in modern representatives (Bernhard and Sen Gupta 2003; Koho and Piña‐Ochoa 2012; Glock 2023). Benthic Foraminifera exhibit a wide variety of mechanisms to deal with low oxygen availability, including morphological adaptations such as their calcitic test shape (Bernhard 1986), thickness (Bernhard 1986; Harman 1964), or porosity, that is, area covered by pores connecting the cytoplasm and the surrounding seawater through the test (Richirt et al. 2019; Kuhnt et al. 2013; Glock et al. 2011), and metabolic adaptations such as dormancy (Ross and Hallock 2016), kleptoplasty (Bernhard and Bowser 1999; Pillet et al. 2011), endobionts and ectobionts hosting (Bernhard et al. 2001; Bernhard 2003; Bernhard, Edgcomb, et al. 2012; Bernhard et al. 2010), fermentation (Orsi et al. 2020; Gomaa et al. 2021), peroxisome proliferation (Bernhard and Bowser 2008) or denitrification (Risgaard‐Petersen et al. 2006). Recently, Foraminifera have been recognized as one of the few eukaryotic groups that can denitrify (Risgaard‐Petersen et al. 2006), and numerous benthic species have been shown to perform denitrification (Piña‐Ochoa, Høgslund, et al. 2010). However, genomic analyses have suggested that the denitrification pathway in Foraminifera may be incomplete (Orsi et al. 2020; Gomaa et al. 2021; Woehle et al. 2018), possibly being completed by the activity of Desulfobacteraceae present in their microbiome (Woehle et al. 2022). Benthic Foraminifera can be responsible for a significant fraction of the total benthic denitrification in low‐oxygen environments (e.g., Skagerrak, Bay of Biscay, and Oxygen Minimum Zone [OMZ] Chile (Piña‐Ochoa, Høgslund, et al. 2010), OMZ Peru (Glock et al. 2013), Santa Barbara basin (Bernhard, Casciotti, et al. 2012), Yellow Sea (Xu et al. 2017)), and even up to 100% in oxygenated parts of the Swedish Gullmar Fjord (Choquel et al. 2021). Consequently, they represent major protagonists in this process and play a key role in the global marine nitrogen (N) cycle. Yet, relatively little is known about the mechanisms involved in foraminiferal denitrification.

While NO_3_^−^ accumulation has been reported in a wide variety of eukaryotic microbes, for example, diatoms, gromiids, fungi, dinoflagellates, haptophytes, chlorophytes, and foraminifers, the intracellular compartment responsible for NO_3_^−^ storage is still unknown (Kamp et al. 2015). Intracellular NO_3_^−^ storage is a widespread phenomenon in Foraminifera, sometimes with concentrations several thousand times higher than in their surrounding environment (Risgaard‐Petersen et al. 2006; Piña‐Ochoa, Høgslund, et al. 2010). Additionally, a remarkably developed vacuolar system was observed in denitrifying species (Bernhard, Edgcomb, et al. 2012; Bernhard, Casciotti, et al. 2012; Nomaki, Toyofuku, et al. 2015; Nomaki et al. 2018). These vacuoles, appearing empty when observed with Transmission Electron Microscopy (TEM) images, were sometimes found to be surrounded by denitrifying endobionts (Bernhard, Edgcomb, et al. 2012; Bernhard et al. 2006) or mitochondria and peroxisome clusters (Bernhard and Bowser 2008), organelles involved in NO_3_^−^ respiration metabolism (Gomaa et al. 2021; Powers et al. 2022). These observations led to the hypothesis that vacuoles are likely the place where NO_3_^−^ is stored in the cell (Bernhard, Edgcomb, et al. 2012; Woehle et al. 2018), as observed in certain bacteria (*Thioploca* (Fossing et al. 1995), *Beggiatoa* (McHatton et al. 1996)) or plants (De Angeli et al. 2006). Generalizing this assumption, (Woehle et al. 2018) proposed a mechanistic model for denitrification in foraminifers of the genus *Globobulimina*, based on genomic and transcriptomic analyses. According to this model, mitochondria perform denitrification and may use the NO_3_^−^ (1) stored intracellularly in vacuoles or (2) directly extracted from the individual's surrounding environment, with NO_3_^−^ carriage between these compartments being mediated by NO_3_^−^ transporters. The involvement of mitochondria in the foraminiferal denitrification pathway is also indicated by (Powers et al. 2022). Understanding the mechanisms of intracellular NO_3_^−^ storage and utilization is crucial because Foraminifera may represent a substantial NO_3_^−^ pool in the sediment that can be transported and released in the sedimentary column or in the overlying water (as N_2_ after denitrification or as NO_3_^−^ if the individual dies), thereby exerting a significant influence on N‐cycling (Glock 2023; Kamp et al. 2015; Dale et al. 2016; Xu et al. 2021). Currently, vacuolar storage of NO_3_^−^ in Foraminifera remains speculative and requires validation to comprehend how this reservoir plays a role in the marine N‐cycle.

Beyond denitrification linked to the N‐cycle, recent gene expression analyses suggest that foraminifers thriving in anoxic conditions possibly use the phosphagen creatine phosphate to quickly regenerate ATP, being an alternative metabolic pathway to couple the energy producing and consuming processes in the cell, and to enable energetically demanding activities under anoxia (Orsi et al. 2020). Some benthic foraminiferal species from the Peruvian OMZ were also shown to accumulate dissolved inorganic phosphorus (DIP, polyphosphate form) inside their cell with concentrations 100–1000 times higher than their surrounding environment (Glock et al. 2020). Pointing out its probable significance for the oceanic P‐cycle, (Glock et al. 2025) showed that phosphate (PO_4_^3−^) storage was occurring in benthic foraminifers distributed in different habitats from tidal mudflats to oxygen‐depleted deep sea, within organelles supposedly identified as acidocalcisomes (Glock et al. 2020; Glock et al. 2025). Acidocalcisomes are organelles that are widely distributed across life, from bacteria to humans, and exhibit various sizes, shapes, abundances, and elemental content, with this heterogeneity supposed to be species‐specific (Docampo et al. 2005). They have various roles in the cell, such as storage of phosphorus (P) compounds and cations (i.e., Mg^2+^ and Ca^2+^), intracellular pH homeostasis, and osmoregulation (Docampo et al. 2005; Docampo and Moreno 2011), or heavy metals detoxification by sequestering and transporting these toxics out of the cell (e.g., nickel, cadmium, lead (Docampo 2006; Keasling 1997; Kulaev et al. 1999)).

To date, the precise interactions between oxic respiration, denitrification, and DIP metabolism of Foraminifera are still unclear. To unveil these relationships, we selected the benthic foraminifer *Bolivina spissa*, which is commonly found in deep‐sea oxygen‐depleted environments and accumulates NO_3_^−^ in its cell (Glud et al. 2009; Nomaki, Chikaraishi, et al. 2015) to perform denitrification (Glock et al. 2019). The species also exhibits a distinct and large vacuolar system (Nomaki et al. 2018). *Bolivina spissa* was even suggested to prefer NO_3_^−^ respiration over oxygen respiration to sustain its metabolism (i.e., facultative aerobe (Glock et al. 2019)), and thus represents a very good candidate species to investigate NO_3_^−^ storage in vacuoles. Furthermore, the presence of organelles identified as putative acidocalcisomes, which have never been described in Foraminifera before, was reported very recently in *B. spissa* (Glock et al. 2025).

Conventional TEM imaging has been used for decades to investigate the ultrastructure of Foraminifera, including test structures, specific organelles, and endobionts/ectobionts (Bernhard and Geslin 2018). However, conventional sample preparation methods, which involve dehydrative fixation through water‐resin exchange, can alter the composition or lead to the loss of water‐soluble elements such as NO_3_^−^. Consequently, the technical challenge resides in visualizing the distribution of dissolved compounds within the ultrastructure. One way to preserve the liquid phase is the rapid cryo‐fixation of the specimen, which maintains the cell undamaged and fully hydrated and allows for the exposure of internal parts using freeze‐fracture or cryo‐sectioning. However, this technique is challenging to implement for Foraminifera, due to their relatively larger size in contrast to other protists or bacteria, and the need to “open” the test to expose the cell content and the composition of finer structures (e.g., organelles (Okada et al. 2024)). At present, only a few recent studies have investigated the soluble intracellular content using cryo‐scanning electron microscopy (SEM) coupled with energy dispersive X‐ray spectroscopy (EDS) (Glock et al. 2025; Okada et al. 2024; Khalifa et al. 2016, 2018; Dubicka et al. 2023), primarily due to the complexity of the procedure (Okada et al. 2024; Goldstein and Richardson 2002). Furthermore, the study of deep‐sea species, such as *B. spissa* , which should preferably be fixed rapidly after sampling (in most cases on‐board), adds a supplementary practical constraint.

To overcome these difficulties and assess the elemental distribution colocalized with organelles within the cell, we employed our newly developed method designed for Foraminifera samples. This method enables the mapping of intracellular soluble compounds on cryo‐fixed specimens using cryo‐SEM‐EDS (Okada et al. 2024). It allows us to concurrently (1) image the intracellular content to identify organelles and (2) map the elemental distribution inside the cytoplasm including the soluble contents. The colocalization of ultrastructure and elemental mapping will indicate the elemental composition of the identified organelles.

This study aims to answer the following questions:

- Are vacuoles the storage organelle of NO_3_^−^ in the cell of *B. spissa* ?
- If NO_3_^−^ is stored in vacuoles, does the NO_3_^−^ concentration (or presence/absence) in the vacuoles change according to the electron acceptor type (O_2_ and NO_3_^−^) and concentrations in the surrounding environment of *B. spissa* ?
- Can we identify putative acidocalcisomes, organelles potentially involved in P storage, in other *B. spissa* specimens?

To answer these questions, *B. spissa* individuals sampled from the Sagami Bay (Japan, 1410 m water depth) were cryo‐fixed onboard directly after sampling or after 48 h of incubation in the presence and/or absence of O_2_ and NO_3_^−^. Additional specimens were fixed with glutaraldehyde for comparison with conventional TEM imaging. The analysis of cryo‐SEM images combined with their complementary EDS maps will allow us to access the ultrastructure in association with the elemental composition, including the soluble phase. The study of specimens incubated in different experimental conditions will allow us to evaluate potential changes in ultrastructure and elemental distribution/composition in organelles regarding the availability of electron acceptors (O_2_ and NO_3_^−^) in the media.

## 2 Material and Methods

### 2.1 Sampling Sites

Using the deep submergence research vehicle *Shinkai 6500* on‐board the Research Vessel *Yokosuka*, sediment cores (8.2 cm in diameter) were sampled using a push core from the central part of the Sagami Bay at 1410 m water depth (NSB site, 35°00.3′ N 139°22.7′ E), during three sampling campaigns in May and October 2022 and May 2023 (Table 1).

**TABLE 1:** Sampling period, sediment interval of sampled specimens, type of analysis performed, fixation type and timing (directly onboard after sampling or after the experiment), and number of specimens analyzed.

Sampling period	Sediment interval	Type of analysis	Fixation type	Fixation timing	Number of specimens analyzed
May 2022	Every cm from 0 to 4 cm	TEM	4% Glutaraldehyde	After sampling	8
October 2022	0–1 cm	Cryo‐SEM‐EDS	Cryo‐fixed	After sampling (“field” specimens)	2
October 2022	0–1 cm	Cryo‐SEM‐EDS	Cryo‐fixed	After experiment	13
May 2023	0–1 cm	Cryo‐SEM‐EDS	Cryo‐fixed	After sampling (“field” specimens)	1
May 2023	0–1 cm	Cryo‐SEM‐EDS	Cryo‐fixed	After experiment	6

### 2.2 Samples for TEM Imaging

The sediment core dedicated to TEM analyses was sliced every centimeter from 0 to 4 cm depth directly after sampling. Samples were processed following previously published protocols for TEM sample preparations (Nomaki, Toyofuku, et al. 2015; Nomaki et al. 2018). Sediment samples were then fixed with 4% glutaraldehyde onboard directly after slicing. In the laboratory on land, samples were gently washed by removing finer sediment through decantation using artificial seawater at 4°C. Only *B. spissa* specimens considered to be living at the time of fixation (based on cytoplasm color and presence of sediment around the aperture) were isolated under a stereomicroscope. Specimens were then embedded in 1% agarose in aqueous sucrose solution and cut into ~1 mm cubes. Samples were decalcified with 0.2% ethylene glycol tetraacetic acid (EGTA) in 0.81 mol L^−1^ aqueous sucrose solution (pH 7.0), rinsed with filtered seawater, and postfixed with 2% osmium tetroxide in filtered seawater for 2 h at 4°C. Samples were rinsed with an 8% aqueous sucrose solution and stained with 1% aqueous uranyl acetate for 2 h at room temperature, then rinsed with reverse osmosis water, dehydrated in a graded ethanol series, and embedded in epoxy resin. The resin blocks were further sectioned into semi‐thin sections (0.5 μm) and ultra‐thin sections (60 nm) using a diamond knife (Diatome) and an ultramicrotome (UC7, Leica). Semi‐thin sections were mounted on glass slides, stained with 1% toluidine blue, and observed under a stereomicroscope (Olympus BX51). Ultra‐thin sections were mounted on a formvar‐supported copper grid mesh and subsequently stained with 2% aqueous uranyl acetate and lead stain solution (0.3% lead nitrate and 0.3% lead acetate Sigma‐Aldrich). TEM observations were performed with a bottom‐mounted 2 × 2 k Eagle charge‐coupled device (CCD) camera (Tecnai G2 20, Thermo Fisher Scientific) at an acceleration voltage of 120 kV. Eight specimens were examined in total (Table 1).

### 2.3 Samples and Experimental Setup for Cryo‐SEM Fixation

For cryo‐SEM‐EDS analyses, living specimens of *B. spissa* (as described earlier) were isolated from the topmost centimeter of the sediment core using a glass pipette under a stereomicroscope onboard within the day of sampling. Specimens were kept at 4°C, mirroring their natural environment throughout the whole procedure.

Some specimens were cryo‐fixed immediately after isolation as described in (Okada et al. 2024) during the October 2022 and May 2023 campaigns and will be referred to hereafter as “field” specimens. Part of the cryo‐SEM‐EDS analyses of these field specimens was previously published in (Glock et al. 2025) for phosphorus (P), calcium (Ca), and magnesium (Mg) elements. Additional specimens were dedicated to two experiments conducted in October 2022 and May 2023 onboard Research Vessel *Yokosuka*. These experiments consisted of incubations of *B. spissa* specimens in varying O_2_ (saturated O_2_ hereafter referred to as oxic, and anoxic) and NO_3_^−^ concentrations (40 and 0 μM).

Prior to each experiment, individuals were acclimated in oxic conditions and the presence of NO_3_^−^ for 24 h at 4°C in the dark. Specimens were placed in a glass Petri dish (8 cm in diameter) filled with filtered bottom sea water from the sampling site (~40 μM of NO_3_^−^ (Glud et al. 2009)), which was previously oxygenated by shaking it vigorously with ambient air and kept at 4°C.

After acclimation, five individuals of *B. spissa* were put in plastic Petri dishes (5 cm in diameter) previously filled with 5 mL of the mixes prepared as follows:

- NO_3_^−^ free seawater from north‐western Pacific (NO_3_^−^ and NO_2_^−^ < 0.1 μM, measured with colourimetric method) was used for the experimental incubations in the absence of NO_3_^−^.
- For incubation in 40 μM NO_3_^−^, the NO_3_^−^ free seawater was amended with KNO_3_ to achieve the desired concentration.

Both mixes were then vigorously shaken with ambient air just before the start of the experiment to reach oxygen saturation. In each Petri dish, a few droplets of autoclaved sediment < 32 μm (from the sampling location) were added as a food source. In the first experiment conducted in October 2022, anoxia was achieved using Anaeropack (Thermo Fisher NRE4650) which converted O_2_ into CO_2_ within the sealed tank. However, the individuals incubated in this first experiment showed significant decalcification upon subsequent observation, which was caused by an unintentional decrease in pH in seawater in anoxic conditions. To address this issue, we conducted a second experiment in May 2023, in which N_2_ gas was continuously flushed into the tank, maintaining positive pressure controlled throughout the experiment to ensure anoxia. In this second experiment, the absence of O_2_ in the tank was confirmed by oxygen optode (Presens) measurements a few minutes after the start of the experiment. Specimens from this second experiment did not exhibit decalcification upon later observation.

**FIGURE 1:**
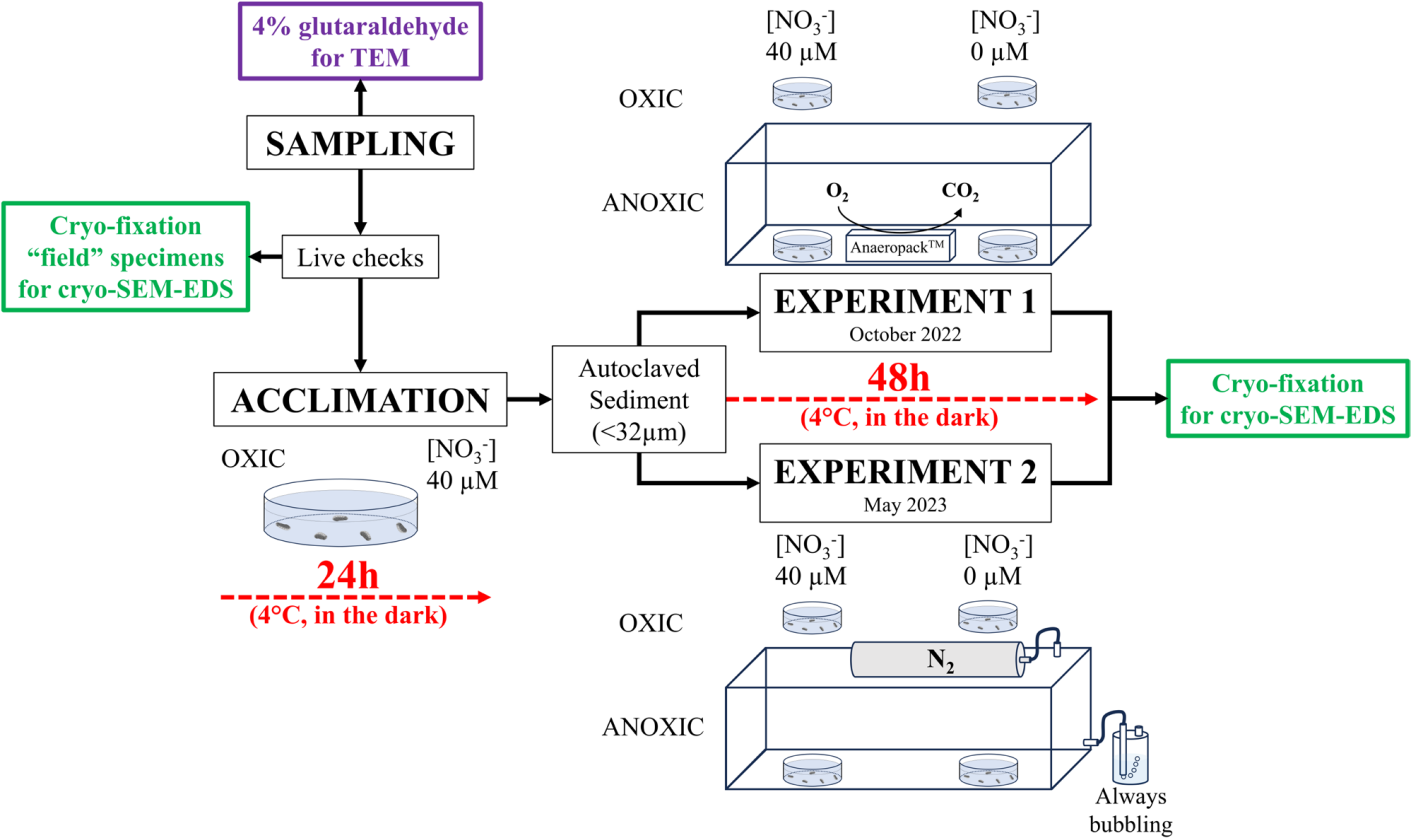
Scheme representing the onboard workflow. The difference between experiments 1 and 2 is the method used to achieve anoxia: for experiment 1, Anaeropack was used to turn O_2_ to CO_2_ in the sealed tank. For experiment 2, N_2_ was continuously flushed, and positive pressure inside the tank was controlled using a bubbling device.

For both experiments, individuals were incubated in the experimental conditions for 48 h in the dark at 4°C. At the end of the experiment, specimens were embedded in a sucrose‐based aqueous glue and cryofixed (in liquid nitrogen‐cooled isopentane) as quickly as possible (within a few minutes (Okada et al. 2024; Richirt et al. 2024)). Specimens were then stored at ~ −170°C until disembarkation (Okada et al. 2024). The schematic workflow conducted onboard for both experiments is summarized in Figure 1.

In total, 22 individuals were cryo‐fixed for subsequent cryo‐SEM imaging and EDS elemental mapping. We aimed to obtain results for ~3 specimens per condition because the sample preparation, measurements, and analyses requires considerable time for this novel method. Among these 22 specimens, three were cryo‐fixed directly after sampling (field specimens (Glock et al. 2025)) and 19 specimens were cryo‐fixed at the end of incubation experiments (Table 1, Table S1). In experiment 2 (anoxia reached by N_2_ flushing), only specimens from anoxic conditions were analyzed to evaluate a potential pH effect in experiment 1 (anoxia reached using Anaeropack).

### 2.4 Cryo‐SEM Imaging, EDS Maps Acquisition, and Post‐Treatment

In the laboratory on land, cryo‐fixed specimens were cracked open in a cryo‐ultramicrotome (Ultracut S equipped with FCS, Leica) using a diamond knife aiming for a clean cut to obtain a flat sample surface. Cryo‐SEM imaging was performed on a Helios G4 UX (Thermo Fisher Scientific) equipped with a gallium focused ion beam (FIB) gun, an EDS detector (Octane Elite Super C5, AMETEK), and a cryogenic stage with a preparation chamber (PP3010T, Quorum). After sublimation of the overlying water ice (~5 min at ~ −80°C) to expose ultrastructure, chromium was sputter‐coated in the preparation chamber (20 mA, 30 s) to suppress charging artifacts. Chromium was chosen instead of conventional gold or platinum to prevent overlap between the K‐ (5.414 keV) and L‐lines (0.573 keV) from the coating and the K‐lines of interest, that is, N (0.392 keV) and P (2.013 keV). After coating, several SEM images were acquired and tiled to obtain a single high‐resolution image for each individual (backscattered electron, BSE mode). Elemental composition was then mapped by EDS analysis for different regions of interest. The acquired X‐ray intensities were corrected by the atomic number‐absorption‐fluorescence (ZAF) method (Castaing 1951), and the bremsstrahlung effect was minimized using the total counts (see the exact procedure in (Glock et al. 2025; Richirt et al. 2024)). Colocalisation of SEM images and EDS elemental maps was performed manually using the clearly visible tests on SEM images and Ca‐EDS maps, calcite being the main compound of the test of *B. spissa* . Image upscaling and/or downscaling was applied when necessary but always without distortion. Finally, a median filter with a radius of 1 μm was applied to each pixel of all the elemental maps used in this study. The workflow performed to colocalise SEM and EDS maps using the Ca element and the EDS maps processing from the original acquisition is presented in Figure S1. Because the pixel intensities in EDS maps are proportional to the imaging time as well as the sample's elemental density, only ratios between elements or between different regions of interest for a given map (i.e., not between different EDS acquisitions) can be calculated. We considered the sucrose‐based glue used to embed specimens before cryo‐fixation as the reference background noise for each element (i.e., absence of the element considered (Okada et al. 2024)).

To evaluate whether vacuoles were N‐enriched, the following procedure was applied:

1. The averaged gray values (per pixel) of three regions representative of the cytoplasm (i.e., without vacuole structures on SEM images) were measured on the corresponding EDS map, then the average (*μ*_ref_) and standard deviation (sd_ref_) of these three reference regions were calculated.
2. The averaged gray value (per pixel) of each vacuole was measured on the corresponding EDS map after colocalization with SEM images.
3. If the averaged gray value of a vacuole was > *μ*_ref_ + 3sd_ref_, the vacuole was considered N‐enriched. If the averaged gray value of a vacuole was < *μ*_ref_ + 3sd_ref_, the vacuole was considered not N‐enriched.
4. The enrichment factor for each vacuole was calculated as the ratio between the averaged gray value of the vacuole and the corresponding *μ*_ref_ value for that specific vacuole.

Enrichment factors given for other elements were estimated using a similar method.

Among the 22 specimens analyzed, five were deemed unsuitable to analyze due to the poor overall quality of the sample in SEM images, resulting from the numerous challenging preparation steps (based on several criteria, see Table S1, specimens field‐B, oxic40‐B, anoxic0‐A, anoxic0‐B, and anoxic0‐D). One additional specimen (anoxic0‐G) was excluded because the corresponding EDS map showed a central dark area, making it unsuitable for elemental analyses (Table S1). These six specimens were excluded from further analyses. Their SEM images and EDS maps, along with those of the 16 other specimens analyzed in this study, are available in Supplementary Plates.

## 3 Results

### 3.1 Identification of Organelles

The ultrastructural examination of the 16 specimens using cryo‐SEM images revealed structures within the cytoplasm of *B. spissa* exhibiting various textures, shapes, sizes, and contrasts (indicative of different elemental composition). Data should be interpreted with caution since the number and size of these structures observed on SEM images may depend on the cross‐section position and angle within the individual and the total surface of exposed cytoplasm (given in Table S1).

**FIGURE 2:**
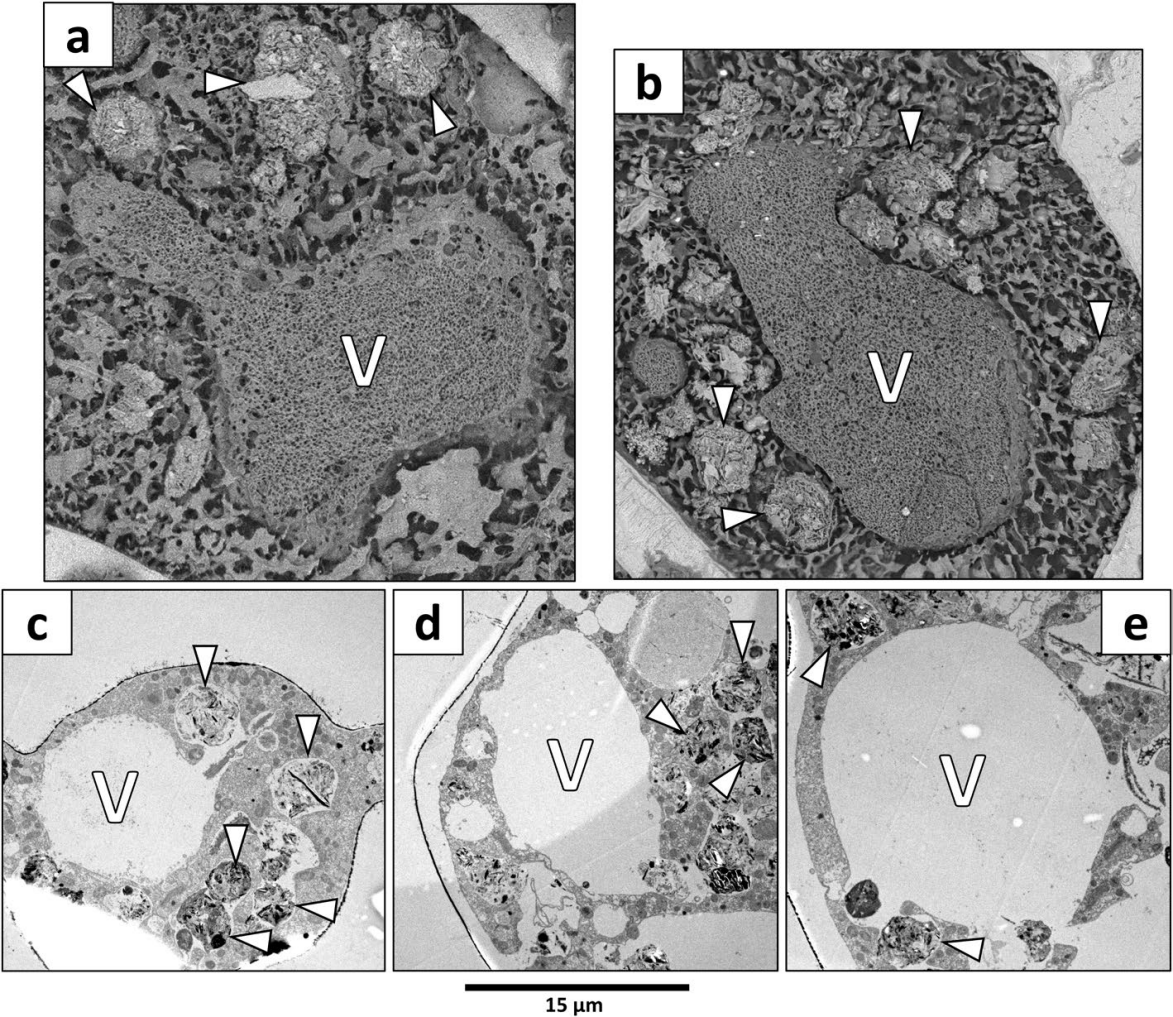
(a, b) Cryo‐SEM images of *B. spissa* (ind. oxic40‐A) showing vacuoles (V) with a homogeneous texture and a more densely meshed network compared to the surrounding cytosol. White arrowheads indicate degradation vacuoles (sensu (LeKieffre et al. 2018)), having a rounded shape and filled with heterogeneous material. Note the broken piece of diatom frustule in the top digestive vacuole on b. (c–e) TEM images of *B. spissa* showing empty vacuoles (V) and degradation vacuoles (white arrows). All images are scaled.

The largest identifiable structures had sizes ranging from 3 to 37 μm in their longer dimension (Table S2) and exhibited a homogeneous texture with a more densely meshed network compared to the surrounding cytosol (Figure 2a,b). These organelles exhibited a generally circular shape with rounded outlines and had morphology, size, and abundance similar to those of the empty vacuoles observed in TEM images (Figure 2c–e). Consequently, these structures were identified as vacuoles. On the 16 specimens examined, 233 vacuoles were identified in 13 specimens, while no vacuoles were identifiable in three specimens (Table S2).

**FIGURE 3:**
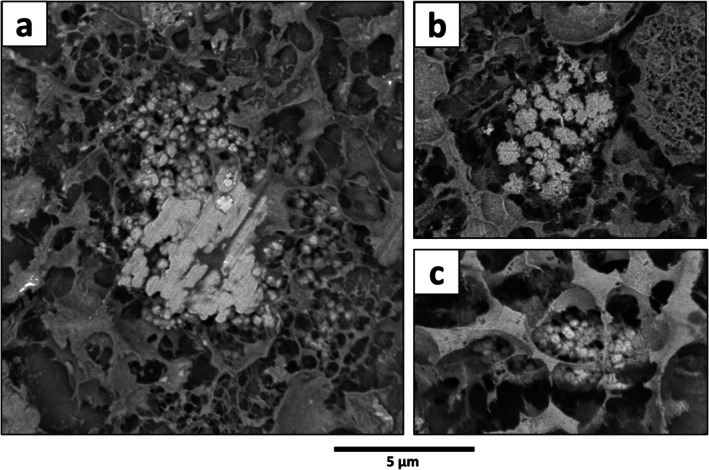
(a–c) SEM images of potential acidocalcisomes, exhibiting a granular texture, found in the cytoplasm of three different individuals of *B. spissa* (oxic40‐C, oxic40‐A, and anoxic40‐C for a, b, and c, respectively).

Granular structures with size ranging from 3 to 19 μm in their longer dimension and appearing brighter than the surrounding cytosol on SEM images (i.e., whitish) were identifiable (Figure 3a–c). These structures predominantly displayed a rounded general outline and were composed of several small, rounded granules of approximately 200–400 nm in diameter aggregated together (Figure 3a–c). Although these granular structures were not identified on TEM images, they occurred in cryo‐fixed samples observed with SEM in almost all chambers except the proloculus. These organelles were identified as putative acidocalcisomes, as described in (Glock et al. 2025). In total, 86 putative acidocalcisomes were identified in 10 out of the 16 individuals studied (Table S3).

**FIGURE 4:**
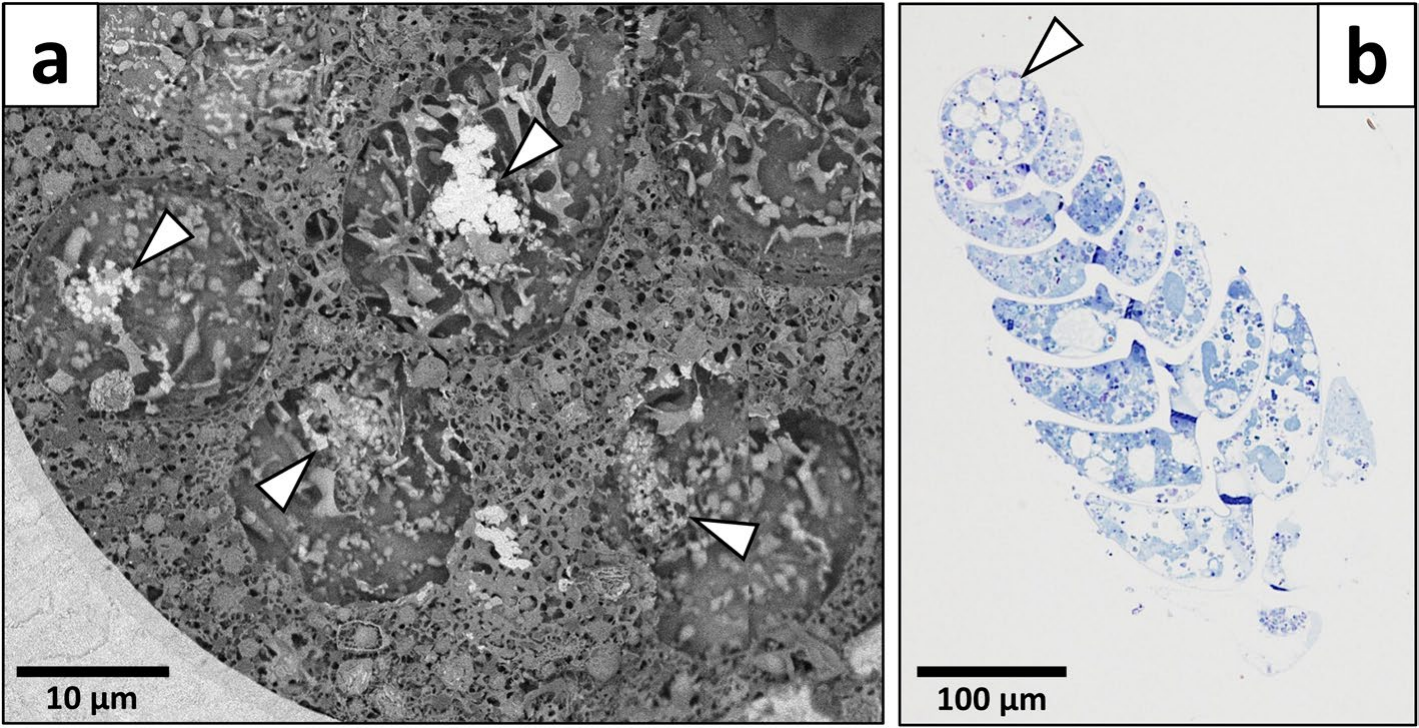
(a) Cryo‐SEM images of the proloculus content showing vesicles with a looser meshed network texture compared to the surrounding cytosol and encompassing whitish granular substructures (indicated by white triangles, ind. oxic40‐A). (b) semi‐thin section of a representative *B. spissa* individual observed under an optical microscope. Note the empty vesicles in the proloculus (indicated by a white triangle).

In specimens for which the proloculus was visible (8 out of 16 individuals, Table S4), characteristic structures only located in the proloculus were visible on cryo‐SEM images. These rounded vacuoles had various sizes ranging from 9 to 33 μm in diameter (Figure 4a, Table S4) and their content exhibited a loosely connected meshed network compared to the surrounding cytosol (Figure 4a). In most cases, a whitish granular structure in a central position and composed of minute granules having sizes ranging from 200 to 600 nm in diameter was observed on cryo‐SEM images (Figure 4a). These proloculus‐vacuoles were also visible on semi‐thin sections prepared for TEM imaging but appeared completely empty when observed under an optical microscope (Figure 4b). A total of 38 of these proloculus‐vacuoles containing a granular substructure were present in 7 of the 8 specimens for which the proloculus was exposed by the cryo‐section (Table S4).

The cellular content on cryo‐SEM images for different individuals appeared variable because the preparation method involves several challenging steps (e.g., cryo‐sectioning and sublimation phase prior to observation), resulting in changes in the appearance of the exposed surface (Figure S2). Apart from calcitic test surface dissolution during the anoxic incubation onboard in experiment 1, no substantial difference in ultrastructure was observed between both experiments.

### 3.2 Elemental Distributions and Associated Organelles

**FIGURE 5:**
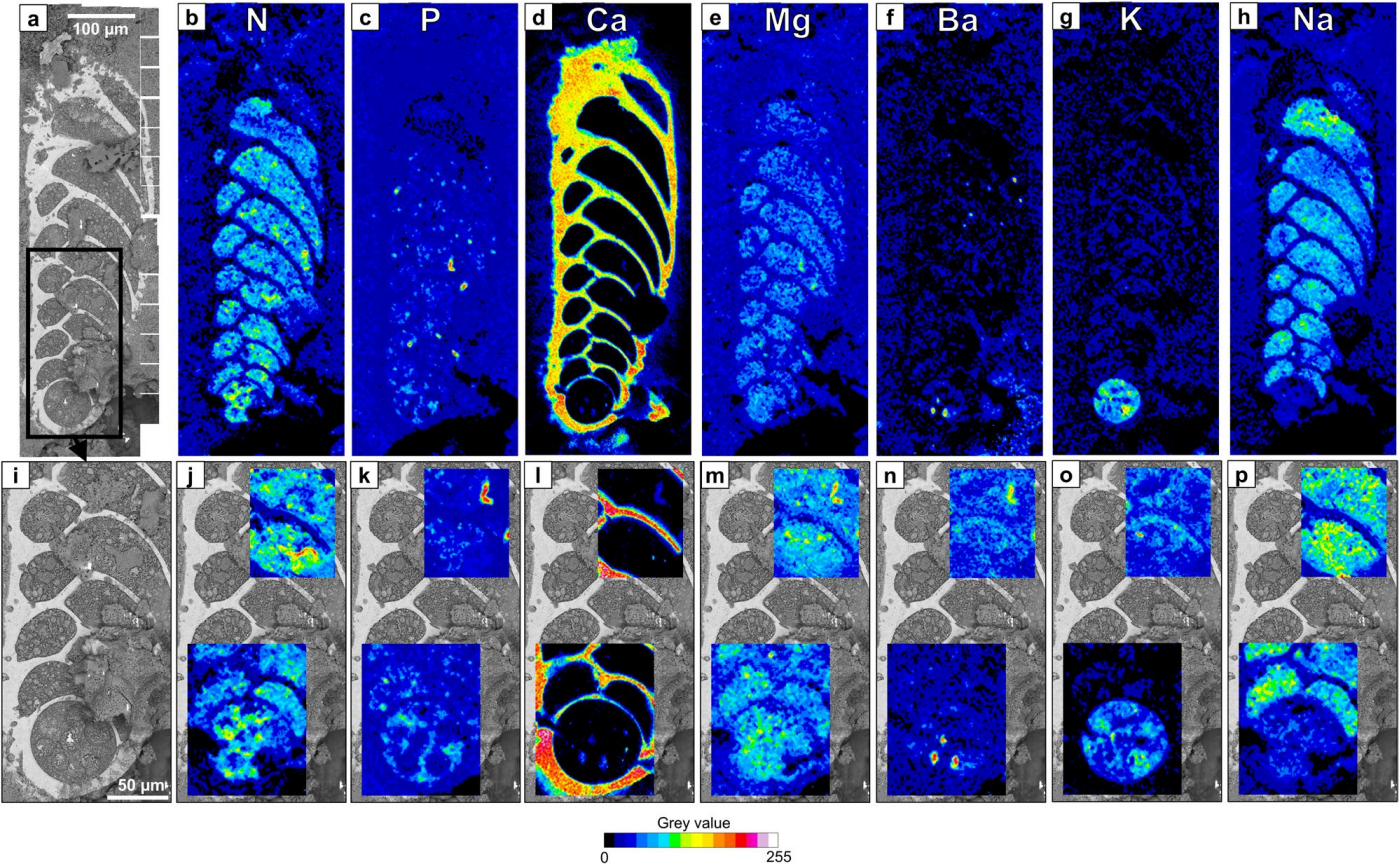
Cryo‐SEM image overview of a representative *B. spissa* individual (from oxic and presence of nitrate, oxic40‐A) with a black square indicating the further magnified area (a) with two regions of interest which were mapped by EDS (i). Elemental distribution for the SEM image overview (b–h) and the regions of interest (j‐p) for N (b and j), P (c and k), Ca (d and l), Mg (e and m), Ba (f and n), K (g and o), and Na (h and p).

Figure 5 presents the overall elemental distributions and magnified mapping of N, P, Ca, Mg, barium (Ba), potassium (K), and sodium (Na) within two regions of interest in the cross‐section of a representative *B. spissa* specimen. All other SEM images and EDS elemental maps for all the specimens analyzed in this study are available in Supporting Information (Supplementary Plates).

**FIGURE 6:**
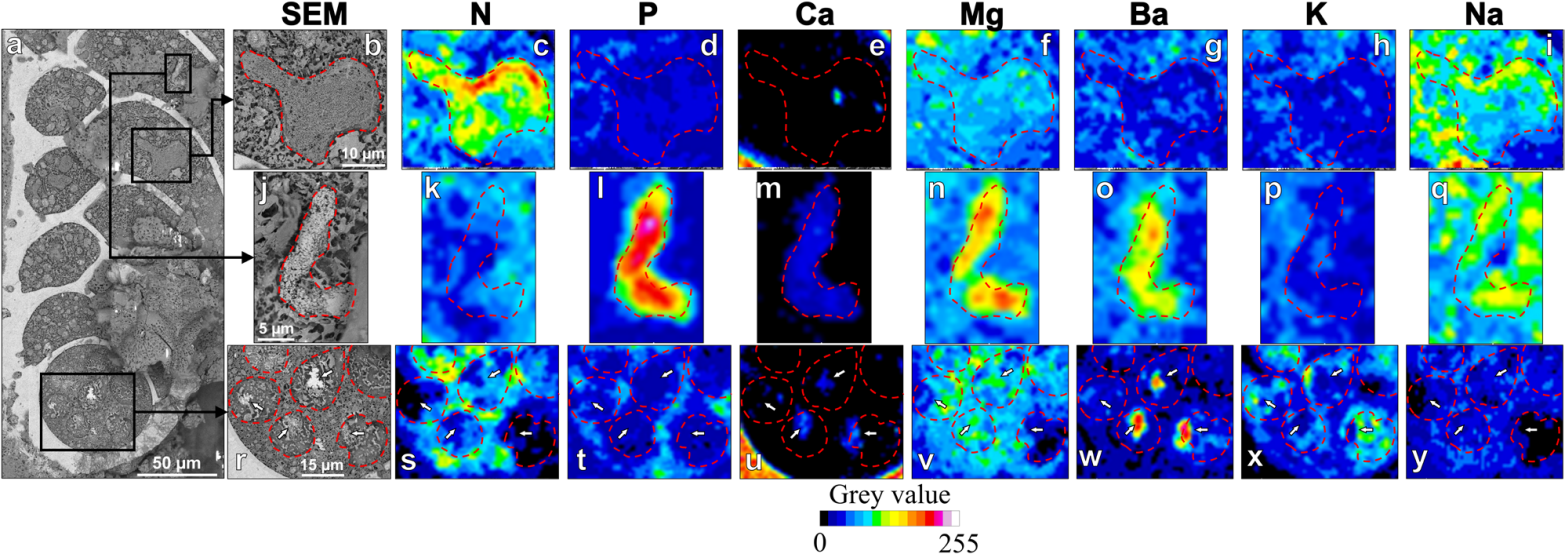
Magnified cryo‐SEM image of regions of interest from Figure 5 (a) with black squares encompassing organelles previously identified. Cryo‐SEM image of a vacuole (red dotted line) (b) and associated EDS map of N (c), P (d), Ca (e), Mg (f), Ba (g), K (h), and Na (i). Cryo‐SEM image of a potential acidocalcisome (red dotted line) (j) and associated EDS maps for N (k), P (l), Ca (m), Mg (n), Ba (o), K (p), and Na (q). Cryo‐SEM image of vacuoles (red dotted lines) containing granular structures (white arrows) located in the proloculus (r) and associated EDS maps for N (s), P (t), Ca (u), Mg (v), Ba (w), K (x), and Na (y).

Nitrogen was distributed almost everywhere in the cytoplasm of *B. spissa* , with some regions being clearly enriched compared to others (Figure 5b,j). Nitrogen‐enriched regions were in most cases colocalized with the vacuoles identified on cryo‐SEM images (Figure 6b–i). Inside these vacuoles, N‐enrichment was sometimes heterogeneous, as emphasized in Figure 6c. In some cases, vacuoles were not N‐enriched compared to the surrounding cytosol (Figure S3, Supplementary Plates).

Phosphorus was mostly accumulated in relatively small regions, while most of the cytosol showed values comparable to those of the sucrose‐based glue (i.e., outside the cell, Figure 5c). The enriched regions corresponded to putative acidocalcisomes on cryo‐SEM images (Figure 6j), which were sometimes also enriched in Ca, Mg, and Ba (Figure 6m–o). Note that the Ca enrichment is probably underestimated because of the strong signal produced by the calcitic test, which masks the signal in the cytoplasmic region for this particular element.

Potassium was mostly accumulated in the proloculus, with all other chambers generally showing comparable values to the external glue (Figure 5g,o, Supplementary Plates). On the contrary, most chambers were enriched in Na compared to the external glue, but the proloculus was depleted in this element compared to other parts of the cytoplasm and showed similar values to the sucrose‐based glue (Figure 5h,p). On magnified EDS maps, the granular structures inside the vacuoles located in the proloculus were Ba‐ and Ca‐enriched (Figure 6u,w, respectively). The vacuole content surrounding these granular structures was generally K‐enriched and Na‐depleted (Figure 6x,y, respectively).

### 3.3 Ultrastructure and Elemental Distribution Changes Regarding the Availability of O_2_ and NO_3_ ^−^

#### 3.3.1 Vacuoles and N

The number and size of vacuoles varied greatly, apparently independently of the individual's origin (fixed directly from the field or incubated in an experiment) and the experimental incubation conditions (presence or absence of O_2_ and 40 μM or 0 μM of NO_3_^−^, Table S2). The specimen with the highest number of vacuoles was incubated under anoxic conditions with the presence of NO_3_^−^ and exhibited a total of 75 vacuoles (Table S2). The three specimens without visible vacuoles were from different origins: one was fixed from the field (field‐C), one was incubated in the presence of O_2_ and NO_3_^−^ (oxic40‐C), and one was incubated in the absence of O_2_ and NO_3_^−^ (anoxic0‐F).

**FIGURE 7:**
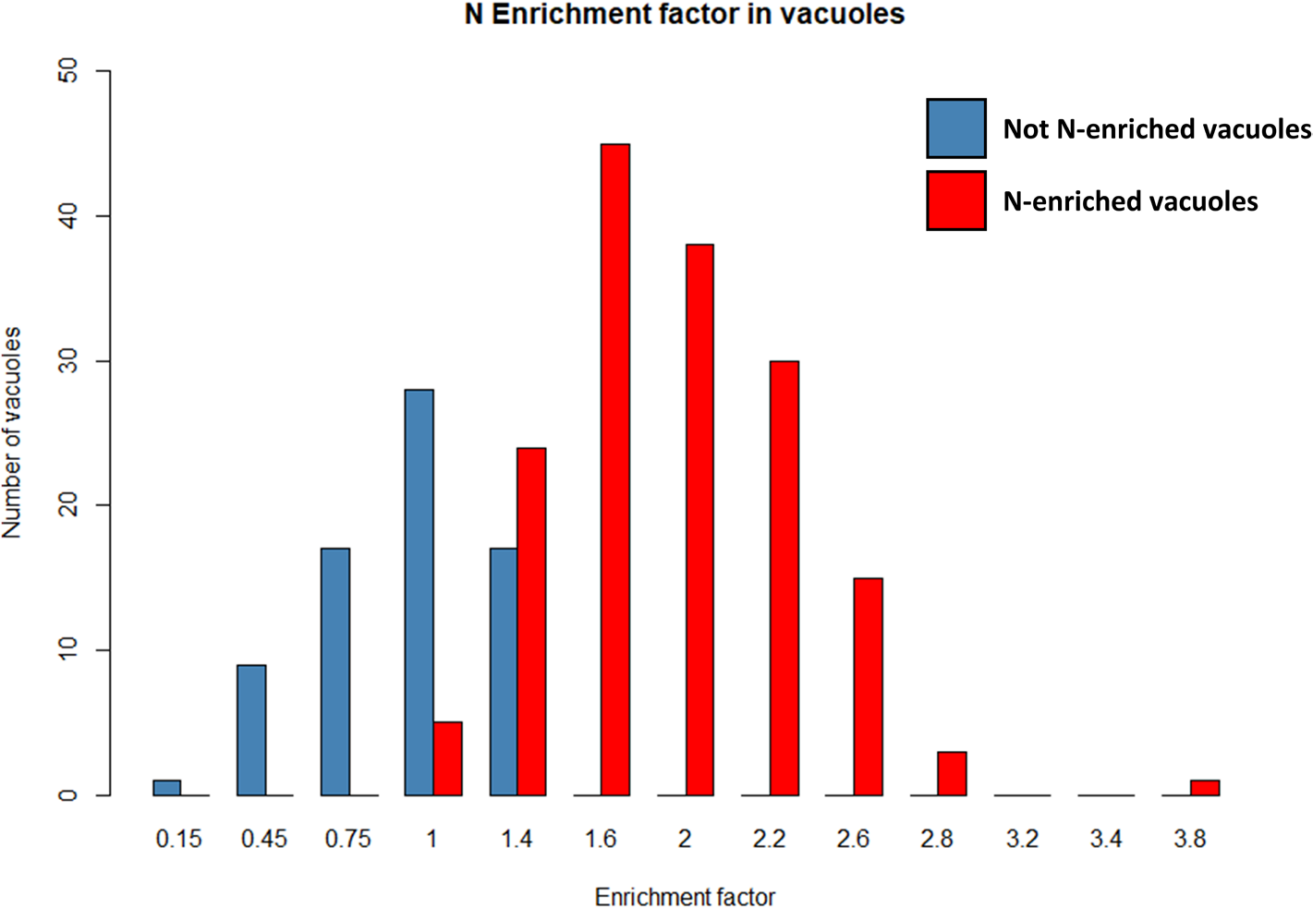
Histogram showing the N‐enrichment factor of vacuoles (see Section 2.4) compared to the surrounding cytoplasm. Vacuoles considered not N‐enriched are in blue and vacuoles considered N‐enriched are in red.

All 13 individuals for which vacuoles were identified on cryo‐SEM showed N‐enriched vacuoles on EDS maps, with most of the specimens (12 out of 13) having the majority of their vacuoles being N‐enriched, with the exception of one specimen (anoxic40‐F) which was incubated in anoxic and presence of NO_3_^−^ conditions (Table S2). In total, over the 233 vacuoles identified, 161 were N‐enriched (69%) and 72 were considered not N‐enriched (31%, Figure 7). In five specimens, all identified vacuoles were N‐enriched compared to the surrounding cytoplasm. These five specimens originated from all conditions (field‐A, oxic0‐C, anoxic40‐B, anoxic40‐C and anoxic0‐E), except the condition where both O_2_ and NO_3_^−^ were present. The N‐enrichment factors measured in vacuoles considered N‐enriched for a given individual ranged from 1.4 ± 0.4 to 2.6 ± 0.6 times compared to the surrounding cytoplasm (Table S2).

Table 2 summarizes the average measurements done for the different experimental conditions and for the field individuals (see Table S2 for individual list). The individuals incubated in the absence of O_2_ and the presence of NO_3_^−^ showed lower enrichment factors in their vacuoles, due to a higher number of vacuoles considered as non‐enriched, especially in a single specimen (Anoxic40‐E, Table S2, Figure S4).

**TABLE 2:** The total number (#) of specimens examined and the number of specimens having visible vacuoles on SEM images for each experimental condition and for field specimens. The average ± standard deviation (SD) of the number of identified vacuole(s) on SEM images, the number of N‐enriched vacuoles on EDS maps, the percentage of N‐enriched vacuole(s) relative to the total number of identified vacuole(s), and the N‐enrichment factor of vacuole(s) (see Section 2.4) are reported for each experimental condition and for field specimens. Average ± SD was calculated only for specimens in which vacuoles were identified. See Table S2 for raw data.

Oxygen (oxic/anoxic)	Nitrate concentration in μM	Specimens examined (#)	Specimens with visible vacuoles (#)	Identified vacuoles on SEM (#)	N‐enriched vacuoles (#)	N‐enriched vacuoles (%)	N‐enrichment factor in all vacuoles
Oxic	40	2	1	20.0	18.0	90.0	1.6 ± 0.2
Oxic	0	3	3	14.7 ± 8.3	12.7 ± 7.2	87.7 ± 12.5	1.8 ± 0.4
Anoxic	40	6	5	23.7 ± 25.6	13.2 ± 8.4	72.0 ± 25.8	1.5 ± 0.6
Anoxic	0	3	3	7.0 ± 7.0	10.0 ± 4.2	96.5 ± 4.9	2.2 ± 0.5
Field (na)	Field (na)	2	1	6.0	6.0	100.0	2.6 ± 0.6

To summarize, no clear and systematic difference between specimens incubated in the different experimental conditions or field specimens was observed (Table 2, Figure S4).

#### 3.3.2 Putative Acidocalcisomes and P/Ca/Mg

Over a total of 16 individuals, 6 individuals did not exhibit potential acidocalcisomes (Table S3). Among them, five were incubated in the absence of NO_3_^−^ (two from oxic and three from anoxic conditions) and 1 in the presence of NO_3_^−^ (anoxic40‐B, Table S3). No putative acidocalcisome was recognized in any of the three individuals incubated in the absence of O_2_ and NO_3_^−^. Most of the presumed acidocalcisomes were P‐enriched for all individuals, with the exception of one individual (anoxic40‐E) incubated under anoxic conditions and the presence of NO_3_^−^ (10 out of 25 were P‐enriched, Table S3). In general, the majority of identified potential acidocalcisomes were P‐enriched (i.e., 62 out of 84, 74%).

Putative acidocalcisomes exhibited a P‐enrichment factor varying from 2 to 7 times depending on the specimen considered, generally being ~3–4 times enriched compared to the surrounding cytoplasm (Figure S5 shows an example of elemental enrichment estimation). They were also ~2–6 times Ca‐enriched in 9 out of 10 specimens (excluding anoxic40‐C, Table S3) and additionally showed in some cases an enrichment in Mg of ~1.5–2 times (7 out of 10 specimens) and Ba of ~2–6 times (5 out of 10 specimens).

#### 3.3.3 Vacuoles in the Proloculus Containing Ba‐Rich Granules

Of 8 specimens for which the proloculus was visible, 7 specimens exhibited 3–7 of these characteristic vacuoles in the proloculus, depending on the specimen considered (Table S4). On a total of 38 proloculus vacuoles identified, 29 were K‐enriched and 31 were Na‐poor compared to the surrounding cytosol. Nineteen of these proloculus vacuoles encompassed a structure composed of granules (such as in Figure 6r) which were Ba‐ and Ca‐enriched in most cases (17 out of 19, 90%).

In all observed proloculi, only one Ba‐rich structure was also S‐rich and was not located within a proloculus–vacuole (ind. anoxic40‐A, Figure S6). Similar Ba‐ and S‐rich particles were sometimes observed outside the proloculus, only in individuals that were incubated in anoxic conditions (but not systematically, i.e., anoxic40‐D, anoxic40‐E, anoxic0‐C, anoxic0‐E, Figure S6). Finally, a single individual exhibited vesicles containing Ba‐rich granular structures without S enrichment outside its proloculus; however, these were not K‐rich (anoxic0‐F, Figure S6).

## 4 Discussion

### 4.1 Organelle Identification Between Conventional TEM and Cryo‐SEM

Conventional electron microscopy preparation involves chemical fixation and dehydration steps, which modify or remove intracellular soluble compounds prior to imaging (Huang and Yeung 2015; Winey et al. 2014). In contrast, fixation by rapid freezing aims to preserve the morphology and composition of native hydrated structures, allowing their subsequent observation and elemental analyses (Hurbain and Sachse 2011). These differences in preparation methods may hamper the direct comparison of intracellular content when observed with both conventional TEM and cryo‐SEM. For instance, it is known that glutaraldehyde fixation and subsequent dehydration may cause significant shrinkage of organelles, up to 15% in linear dimension (~40% in volume (Boyde and MacOnnachie 1979; Mollenhauer 1993)). A striking example of differences between fixation methods (i.e., dehydrated and cryofixed) observed with SEM is given in (Meibom et al. 2023). For Foraminifera, few studies using cryo‐fixation followed by cryo‐SEM imaging often involve methodological differences (e.g., high‐pressure vs. rapid freezing), which complicates comparative organelle identification (Glock et al. 2025; Okada et al. 2024; Khalifa et al. 2016; Khalifa et al. 2018; Dubicka et al. 2023). However, organelle identification in cryo‐SEM samples is undoubtedly facilitated by the many prior TEM studies (e.g., Bernhard, Edgcomb, et al. 2012; Bernhard et al. 2010; Bernhard, Casciotti, et al. 2012; Nomaki et al. 2018; LeKieffre et al. 2018; Goldstein and Richardson 2018).

Overall, a good correspondence was observed between organelles size, shape, and abundance on TEM and cryo‐SEM images for *B. spissa* . Cryo‐SEM observations revealed that the content of the “empty” vacuoles on TEM images is not always the same. At least two different types of vacuoles with a different distribution pattern in the cell could be identified on cryo‐SEM. While one was widespread in the cell except in the proloculus and exhibited a homogeneous texture with a densely meshed network, the other was almost exclusively distributed in the proloculus and showed a more loosely meshed network often containing a granular substructure. The difference between these two types of vacuoles was further confirmed by their contrasting elemental composition on EDS maps. This indicates that structures with a similar appearance on conventional TEM images (i.e., empty vacuoles) may, in fact, correspond to different types of organelles. The cryo‐SEM‐EDS approach also allowed the detection of granular structures in the cytoplasm which were previously identified as putative acidocalcisomes with the same method, based on their elemental compositions, size, and appearance (Glock et al. 2025). Acidocalcisomes were never identified in Foraminifera with any other cell fixation and ultrastructure imaging method to our knowledge. This is likely because acidocalcisomes often appear empty on TEM images depending on the preparation method used, whereas cryo‐techniques are better suited to preserve these structures (Docampo et al. 2005; Docampo 2006).

Cryo‐fixation in this study was effective in identifying organelles with a size > 3 μm. For the exact same preparation procedure, cryo‐SEM image resolution should allow the characterization of structures having a dimension < 1 μm (and for solid material down to 0.05 μm (Richirt et al. 2024)). While numerous smaller structures were visible on cryo‐SEM images, they were difficult to identify with certainty. For instance, mitochondria, peroxisomes, or lipid droplets, which are numerous on TEM preparations of *B. spissa* and often show sizes < 3 μm (LeKieffre et al. 2018), could not be discriminated on cryo‐SEM images. This may be due to the fact that conventional TEM imaging intends to observe membrane structure using different staining methods to identify organelles. In this study, structures detected in the proloculus of one individual had a somewhat similar morphology to peroxisomes, with a circular general shape containing a central structure resembling the catalase core (Bernhard and Bowser 2008) (Figure S7). However, these structures showed a substantial size difference with peroxisomes typically identified in TEM images, being more than three times larger (Bernhard et al. 2010; LeKieffre et al. 2018). This difference cannot be explained only by the potential shrinkage of organelles due to chemical fixation, suggesting instead that they are not peroxisomes.

Finally, several technical challenges emerged during this study and are detailed in Text S1, along with methodological perspectives.

### 4.2 N in Vacuoles

#### 4.2.1 Nitrate Can Be Stored in Vacuoles in *Bolivina Spissa*

This study demonstrates that most of the vacuoles in *B. spissa* are N‐enriched (~2 times) compared to their surrounding cytosol. Since these vacuoles appear empty in dehydrated TEM preparations, the nitrogen they contain must be in a soluble form. Vacuoles may contain soluble N in different forms such as amino acids, ammonium (NH_4_^+^), nitrite (NO_2_^−^), or nitrate (NO_3_^−^) (Raven 1987; Raven 1997). Amino acids are known to be stored in vacuoles in other organisms such as yeast (Bianchi et al. 2019) or plants (Tan et al. 2019). While amino acid fermentation may occur in Foraminifera, as pointed out by a transcriptomic study (Orsi et al. 2020), their intracellular concentration has never been quantified in Foraminifera to our knowledge. Intracellular NH_4_^+^ concentrations were lower than NO_3_^−^ concentrations in different benthic Foraminifera species, as measured by a previous study (Langlet et al. 2020). Furthermore, the congeneric denitrifying species *Bolivina argentea* was shown to express genes coding for enzymes having a low affinity for NH_4_^+^ (glutamine dehydrogenase, GDH (Gomaa et al. 2021)). Additionally, intracellular NO_2_^−^ and NO_3_^−^ concentration measurements performed on the denitrifying species *Globobulimina affinis* (Nomaki, Chikaraishi, et al. 2015) and *B. argentea* (Bernhard, Casciotti, et al. 2012) indicated that NO_3_^−^ was largely predominant over NO_2_^−^ in the cell, especially in *Bolivina*. While we cannot discard other N form storage with certainty, *B. spissa* shows high intracellular NO_3_^−^ concentrations (190 ± 252 pmol NO_2_^−^ + NO_3_^−^ ind^−1^ (Nomaki, Chikaraishi, et al. 2015)), hence we assume that the soluble N enrichment observed in the vacuoles mostly represents NO_3_^−^ in this study. On average, one individual of *B. spissa* contains ~50 ng nitrogen (52 ± 7 ng nitrogen ind^−1^ (Nomaki et al. 2008)) and ~200 pmol NO_2_^−^ + NO_3_^−^ (190 ± 252 pmol NO_2_^−^ + NO_3_^−^ ind^−1^ (Nomaki, Chikaraishi, et al. 2015)). Assuming that NO_2_^−^ + NO_3_^−^ represents mostly NO_3_^−^, ~200 pmol of NO_3_^−^ is equivalent to ~3 ng of N. Based on this estimation, N in the form of NO_3_^−^ in the cell should represent ~10% of the total nitrogen in a single individual. *Bolivina spissa* presents a well‐developed vacuolar system (Nomaki et al. 2018), and ~10% of the total cytoplasmic volume is occupied by vacuoles (Julien Richirt, unpublished). In a hypothetical specimen in which ~10% of the total cellular N content is originating from stored NO_3_^−^ and where ~10% of the total cellular volume is occupied by vacuoles with 70% of them being N‐enriched (as measured in this study, see Figure 7), the N‐enrichment factor of vacuoles compared to their surrounding cytosol measured here (~2 times) is plausible. This approximation must be considered carefully as it may include orders of magnitude of variations, together with the fact that there is no evidence that NO_3_^−^ is absent in other organelles and/or in the cytosol.

Our results are the first to directly show that vacuoles are N‐enriched in *B. spissa* , and strongly support that vacuoles are the NO_3_^−^ storage organelle in the foraminiferal cell. About ~70% of the vacuoles were N‐enriched compared to the surrounding cytosol. The absence of N‐enrichment in other vacuoles may indicate that NO_3_^−^ was either (1) being respired at the time of fixation or that (2) these vacuoles were not dedicated to NO_3_^−^ storage and could be responsible for other unknown function(s). TEM observations indicated that some vacuoles were surrounded by numerous mitochondria and peroxisomes, which are involved in energy metabolism (Bernhard and Bowser 2008), while other vacuoles were not (Figure S8). This finding supports the idea that the vacuoles are possibly not only dedicated to NO_3_^−^ storage and might have additional role(s), requiring further investigation.

#### 4.2.2 Nitrate Storage in Variable Electron Acceptors Conditions

**FIGURE 8:**
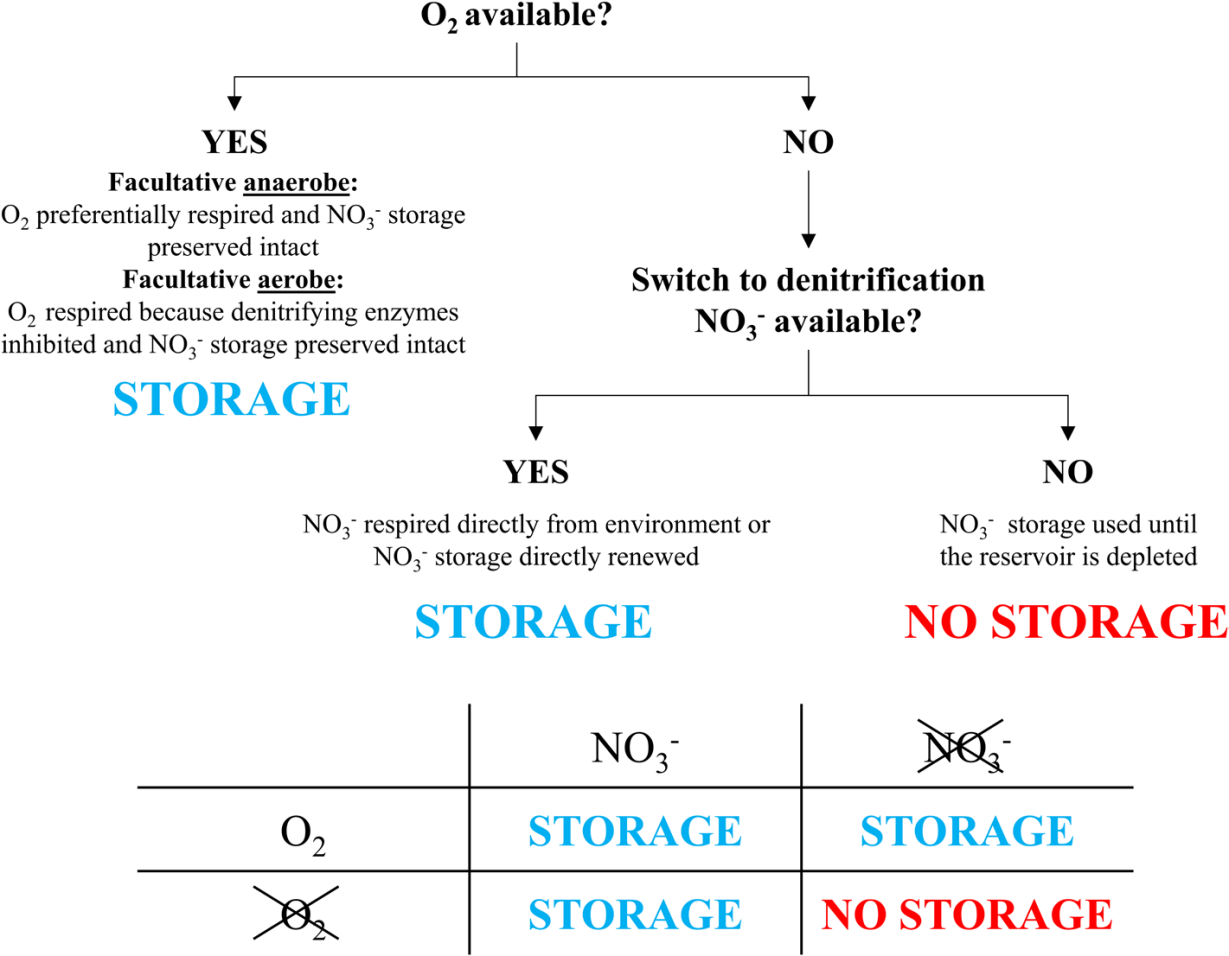
Expected NO_3_^−^ storage response to different electron acceptors availability (O_2_ and NO_3_^−^) for *B. spissa* (based on previous works of (Woehle et al. 2018; Glock et al. 2019; Høgslund et al. 2008; Koho et al. 2011)).

Our experiment was inspired by the model proposed by (Woehle et al. 2018) for *Globobulimina* species, where individuals can uptake NO_3_^−^ from their environment and either (1) use it directly for denitrification or (2) store it in vacuoles for later use when required for metabolic needs. An additional assumption was that individuals can uptake NO_3_^−^ from their environment and store it in vacuoles faster than they consume it for denitrification, such as estimated for other denitrifying species (~10 and ~5 times for *Nonionella stella* and *Globobulimina turgida* , respectively (Høgslund et al. 2008; Koho et al. 2011)). The duration of the experiment in this study, spanning two days, was determined after previous measurements of NO_3_^−^ content (on average ~200 pmol NO_2_^−^ + NO_3_^−^ ind^−1^ (Nomaki, Chikaraishi, et al. 2015)) and denitrification rate (on average ~373 ± 205 pmol N ind^−1^ day^−1^ (Glock et al. 2019)) for *B. spissa* . These values indicate that the average NO_3_^−^ storage of *B. spissa* should allow continuous denitrification for ~0.5 days, hence that 2 days should be enough to consume the NO_3_^−^ intracellular reservoir if no other electron acceptor is available in the individuals' surrounding environment. Figure 8 summarizes the expected NO_3_^−^ storage and use response of *B. spissa* to the presence or absence of an electron acceptor (O_2_ or NO_3_^−^).

After 2 days of incubation, our results showed no clear and systematic difference in the vacuole presence/absence, number, or N‐enrichment factor between the different conditions, thereby preventing any definitive conclusions. The absence of difference in our results may emerge from various reasons including (1) the insufficient number of replicates per experimental condition to clearly identify a pattern, (2) an incomplete understanding of the mechanisms underlying NO_3_^−^ storage and use in *B. spissa* , or (3) the experiment duration was too short. Notably, previously reported denitrification rates and intracellular NO_3_^−^ storage have substantial variability (Nomaki, Chikaraishi, et al. 2015; Glock et al. 2019), a pattern that appears common across the species known to accumulate NO_3_^−^ and denitrify (Glock 2023; Piña‐Ochoa, Høgslund, et al. 2010).

A comparable experiment involving the NO_3_^−^ storing and denitrifying foraminifera *Globobulimina turgida* was conducted by (Piña‐Ochoa, Koho, et al. 2010). In their study, specimens were incubated in the presence and/or absence of NO_3_^−^ and O_2_ for a minimum of 56 days. A high variability in the intracellular NO_3_^−^ pool and turnover was found for individuals incubated in different conditions (Piña‐Ochoa, Koho, et al. 2010). The authors postulated that this variability may originate from the individuals' history of exposure to O_2_ and NO_3_^−^, and potentially food availability (Koho et al. 2011; Piña‐Ochoa, Koho, et al. 2010). Assimilation of NO_3_^−^ from food sources is possible (i.e., directly from organisms containing NO_3_^−^ via predation or through a hypothetical oxidation of NH_4_^+^, that is, nitrification, from mineralized organic matter to NO_3_^−^ when O_2_ is available). However, the pore density of *B. spissa* exhibits a strong correlation with bottom water NO_3_^−^ concentration, suggesting that *B. spissa* primarily uptakes NO_3_^−^ from the surrounding pore water (Glock et al. 2011; Woehle et al. 2018; Govindankutty Menon et al. 2023).

While significant variability in NO_3_^−^ storage and denitrification rate is less likely in a laboratory experiment where individuals are incubated in controlled conditions, it is especially plausible in natural settings (as observed in specimen field‐A and field‐C, Table S2, Supplementary Plates). In nature, individuals may be exposed to variable concentrations of O_2_ and NO_3_^−^ for various durations due to their ability to migrate vertically in the sedimentary column (Geslin et al. 2004; Pucci et al. 2009; Deldicq et al. 2023). This emphasizes the need for an acclimation period to mitigate the individuals' history of exposure to O_2_ and NO_3_^−^ before studies that carry out experimental incubation.

Additionally, Foraminifera are suspected to be able to perform both aerobic respiration and denitrification in parallel without the need for assembling new protein complexes (Woehle et al. 2018). The capacity to regulate the activity levels of the different enzymes involved in both aerobic respiration and denitrification depending on the availability and concentration of electron acceptors was initially described in a eukaryotic mitochondrion in a fungus (Takaya et al. 2003). Although a comparable hybrid respiration mode has not been demonstrated for foraminiferal mitochondria, the existence and mechanisms of such metabolic adjustments, possibly accounting for the considerable variability in measured denitrification rates, require investigation. Finally, a recent study based on a comprehensive gene expression analysis in the kleptoplastidic foraminifer *Nonionella stella* showed that an array of metabolic pathways with a unique mitochondrial metabolism supports chemolithomixotrophy, pointing out the versatility of foraminiferal metabolisms in the absence of oxygen (Gomaa et al. 2025).

The comprehension of the relationship between the metabolic mode of Foraminifera and their direct environmental conditions is especially important in highly variable environments such as coastal areas (e.g., tide, freshwater inputs, salinity, temperature), but also in deep sea environments, where Foraminifera may cross several redox fronts (from oxic to euxinic) over short distances when migrating vertically in the sedimentary column.

### 4.3 P, ca and Mg in Putative Acidocalcisomes

The possible storage organelle for PO_4_^3−^ in the cell of *B. spissa* from the Sagami Bay (the same “field” specimens as in this study) was for the first time investigated by (Glock et al. 2025), providing evidence for the presence of possible acidocalcisomes in Foraminifera. Our results confirm these previous observations and extend the description of putative acidocalcisomes in additional individuals of *B. spissa* that are often enriched in Ca, Mg (such as in (Glock et al. 2025)), and sometimes in Ba. Similar colocalised intracellular enrichments in P and Ca (and sometimes Mg) were observed in *Uvigerina akitaensis*, another benthic foraminiferal species from the Sagami Bay (Okada et al. 2024), suggesting that this species also contains potential acidocalcisomes.

Among the 16 examined individuals in this study, putative acidocalcisomes were present in 10 of them, confirming their prevalence in foraminifers. In the remaining 6 individuals, of which 5 were incubated in the absence of NO_3_^−^, the concurrent absence of these structures and the presence of N‐enriched vacuoles suggest that the PO_4_^3−^ pool is preferentially used over NO_3_^−^ storage to sustain metabolism (except for the individual anoxic0‐F for which neither vacuoles nor putative acidocalcisomes were identified). Finally, the absence of putative acidocalcisomes in all individuals incubated without O_2_ and NO_3_^−^ further supports the hypothesis that these structures serve as reservoirs for PO_4_^3−^, which is mobilized in the absence of an electron acceptor.

Assuming that *B. spissa* is a facultative aerobe (preference for NO_3_^−^ over O_2_ for respiration (Glock et al. 2019)), polyphosphates would act as an abundant and readily available energy source to sustain metabolism when energy bursts are needed (Orsi et al. 2020). This would be especially true if switching from denitrification to aerobic respiration (or vice versa) required metabolic adjustments (such as assembling new protein complexes). In this specific case, PO_4_^3−^ storage may represent an efficient temporal energy buffer to prevent a rapid fall of ATP concentrations (Orsi et al. 2020), when individuals vertically migrate across redox fronts and environmental conditions are changing abruptly. If this is the case, it would suggest that the PO_4_^3−^ reservoir in Foraminifera is likely dynamic through time, accounting for the high variability in PO_4_^3−^ intracellular concentrations (Glock et al. 2025). This would also explain the lack of clear results regarding the NO_3_^−^ storage in the absence of O_2_ and NO_3_^−^ in the experimental incubations of this study; the NO_3_^−^ storage consumption would be delayed until the depletion of the PO_4_^3−^ reservoir.

Overall, our results on P distribution in the cell are not straightforward, emphasizing that the metabolism of *B. spissa* is probably more complex than previously thought and that more studies are urged to better characterize how the cellular machinery is fueled in denitrifying species.

The total quantity of P present in the cell can be approximated by the Redfield ratio between N and P (16:1). *Bolivina spissa* contains ~50 ng of N (Nomaki et al. 2008), hence ~3 ng of P which converts to ~100 nmol.ind^−1^. The intracellular DIP concentration in *B. spissa* is estimated at ~140 pmol.ind^−1^ (Glock et al. 2020). It means that DIP represents ~0.1% of the total P in the cell. Acidocalcisomes represent about 1%–2% of the total cell volume in protists (Docampo et al. 2005; Docampo and Moreno 2011), which is plausible for Foraminifera regarding the general size and abundance of presumed acidocalcisomes in our *B. spissa* individuals. These figures result in potential acidocalcisomes being about 5–10 times more concentrated in P than the surrounding cytosol, which is again very close to the enrichment factors we measured in our individuals (2–7 times). Additionally, using the estimated cell volume (~4 × 10^6^ μm^3^) and DIP concentration per individual (~140 pmol.ind^−1^ (Glock et al. 2020)), the intracellular DIP concentration is estimated to be ~35 mM (in the range of other species in (Glock et al. 2020)). Assuming that the supposed acidocalcisomes represent 1%–2% of total cell volume in *B. spissa* , these figures result in a DIP concentration of ~1.75–3.5 M. This concentration range agrees with the molar range for acidocalcisomes estimated for other protists (i.e., Trypanosomatids (Docampo et al. 2005)).

Finally, the fact that Ba was accumulated in potential acidocalcisomes in 5 specimens out of 10 is congruent with the supposed role of polyphosphate in heavy metal detoxification, although Ba was not specifically mentioned in previous studies (Docampo 2006; Keasling 1997; Kulaev et al. 1999).

### 4.4 K‐Rich and Na‐Poor Vacuoles Containing Soluble Ba in the Proloculus

In the proloculus of most individuals (7 out of 8 individuals), several K‐rich and Na‐poor vacuoles were observed, often containing a central granular structure enriched in Ba and Ca. This suggests that these vacuoles are common in *B. spissa* proloculus. Their prevalence is further supported by semi‐thin section preparations observed with an optical microscope, where they appeared entirely empty (Figure 4b, Figure S9), further indicating that their content is soluble and was removed during the dehydration step through conventional electron microscopy preparations. In cryo‐SEM images, the central structures within these proloculus vacuoles consisted of numerous minute granules, somewhat similar to the granular structure of the putative acidocalcisomes, but they did not contain any P or Mg.

Barium is incorporated in calcareous foraminiferal tests during calcification, and its content in the test, reflecting ambient dissolved Ba concentrations during calcification, may be used in palaeoreconstruction contexts (Lea and Boyle 1989; Lea and Boyle 1991; de Nooijer et al. 2017). However, to our knowledge, the only described intracellular occurrence of this element in Foraminifera is in Xenophyophore species, which may house barium sulphate crystals (BaSO_4_ or barite, not soluble) at relatively high concentrations (Gooday and Nott 1982; Hopwood et al. 1997). Barite crystals may play a role in statocysts (gravity sensors) due to their high density compared to the rest of the cytoplasm in ciliates (Fenchel and Finlay 1986), or contribute to buoyancy in certain diatoms (Stroobants et al. 1991), but their origin and function in Xenophyophores remain under debate (Gooday and Nott 1982; Hopwood et al. 1997). Barite crystal morphology in Xenophyophores (Gooday and Nott 1982; Hopwood et al. 1997) and in the ocean is well described (Griffith and Paytan 2012; Martinez‐Ruiz et al. 2019), and differs from the morphology of the granular structures observed in the proloculus of *B. spissa* . Additionally, the absence of S co‐occurrence on EDS maps discards the barium sulphate nature of these granules in *B. spissa* , further indicating that the Ba encompassed in these proloculus‐vacuoles is in a soluble form.

Soluble Ba is considered a toxic element for the cell, being a physiological antagonist of K, blocking the K^+^ channels of the Na‐K pump in the cell membranes, thus increasing the active influx and inhibiting the passive efflux of K (Oskarsson 2022). It is interesting to note that in *B. spissa* , these Ba‐containing vacuoles (and the proloculus in general) are in most cases K‐enriched and Na‐depleted compared to the rest of the cytoplasm, suggesting a potentially Ba‐induced ionic imbalance. The distribution of these vacuoles exclusively in the proloculus (with the exception of one individual in this study) suggests that the proloculus has unique functions compared to other chambers and may serve as the site for specific metabolic function(s). Additionally, the absence of pores (as observed in (Richirt et al. 2024)) and the narrow connection with the next chamber make the proloculus one of the most isolated parts of the cytoplasm from the environment. This further suggests that the proloculus may function as a relatively separated metabolic bioreactor (or at least for specific reactions) when compared to other chambers (Hottinger 2000). Alternatively, the proloculus might serve as a waste disposal chamber for ionic Ba; nevertheless, the question of why Ba is not excreted from the cell remains unresolved.

## 5 Conclusion

In this study, we applied a new correlative microscopy methodology on cryo‐fixed specimens of the denitrifying *B. spissa* . The method allows for the simultaneous acquisition of high‐resolution SEM images to identify intracellular structures and EDS elemental distributions including the soluble phase. Our results demonstrate that most of the empty vacuoles observed on TEM images in *B. spissa* are enriched in N, most probably NO_3_^−^, supporting the idea that these vacuoles are the NO_3_^−^ storage organelles in the cell. However, not all vacuoles contained higher N content than the surrounding cytoplasm, suggesting that they could have other function(s) besides NO_3_^−^ storage. Our findings also confirm the prevalence of putative acidocalcisomes in *B. spissa* . We hypothesize that the quickly available energy they contain (polyphosphate) may be preferentially used prior to the intracellular NO_3_^−^ storage when switching between respiration modes (aerobic/anaerobic), possibly because they represent a buffer energy enabling metabolic adjustments. Our observations highlight the presence of a new type of organelle localized in the proloculus, which very likely contains soluble Ba. Concurrently, the proloculus was found to be K‐enriched and Na‐impoverished compared to the rest of the cytoplasm, possibly because of the presence of soluble Ba in these vacuoles. While the role of these organelles is still unknown, their presence suggests that the proloculus may serve specific function(s) in *B. spissa* . This study emphasizes the relevance of cryogenic approaches in the assessment of the intracellular soluble content, the confirmation of the existence of hypothetical organelles, and the characterization of new cellular structures.

## Author Contributions

**Julien Richirt:** conceptualization, formal analysis, funding acquisition, investigation, visualization, writing – original draft, writing – review and editing. **Satoshi Okada:** formal analysis, investigation, methodology, visualisation, writing – review and editing. **Yoshiyuki Ishitani:** investigation, writing – review and editing. **Nicolaas Glock:** writing – review and editing. **Katsuyuki Uematsu:** investigation, writing – review and editing. **Hidetaka Nomaki:** conceptualization, funding acquisition, investigation, project administration, writing – review and editing.

## Ethics Statement

The authors declare that this manuscript is exclusively submitted to EMI. The authors declare that the content and authorship of the submitted manuscript have been approved by all authors, and that all prevailing local, national, and international regulations and conventions, and normal scientific ethical practices, have been respected.

## Conflicts of Interest

The authors declare no conflicts of interest.

## Supporting information


**Data S1:** jeu70044‐sup‐0001‐DataS1.docx.

**Data S2:** jeu70044‐sup‐0002‐DataS2.pdf.

## Data Availability

All the data presented in this study are available in the Supporting Information attached to this manuscript.
