# Supplementary material for: Different Elemental Compositions and Potential Functions of Vacuoles in Bolivina spissa (Foraminifera, Rhizaria) Based on Cryo‐SEM‐EDS Analyses
Source: J Eukaryot Microbiol. 2025 Aug 31;72(5):e70044. doi: 10.1111/jeu.70044 (PMC12399780; doi:10.1111/jeu.70044)
Supplement: Supplementary file 1 — Data S1: jeu70044‐sup‐0001‐DataS1.docx. [file JEU-72-e70044-s001.docx]

**Different elemental compositions and potential functions of vacuoles in *Bolivina spissa* (Foraminifera, Rhizaria) based on cryo-SEM-EDS analyses**

Julien Richirt^1^, Satoshi Okada^1^, Yoshiyuki Ishitani^1^, Nicolaas Glock^2^, Katsuyuki Uematsu^3^ and Hidetaka Nomaki^1^

^1^ SUGAR, X-star, Japan Agency for Marine-Earth Science and Technology (JAMSTEC), 2-15 Natsushima-cho, Yokosuka 237-0061, Japan

^2^ Institute for Geology, University of Hamburg, Bundestrasse 55, 20146 Hamburg, Germany

^3^ Marine Works Japan Ltd., 3-54-1 Oppamahigashi-cho, Yokosuka, Kanagawa, 237-0063, Japan

**SUPPLEMENTARY MATERIAL**

*Supplementary Text 1: Methodological challenges and perspectives*

Numerous technical challenges and methodological biases had to be overcome during this work. Here we quickly outline key points to consider, organised according to the workflow. Rapid cryo-fixation is one of the most effective means to preserve original hydrated cellular structures. Yet, artifacts may also emerge from this fixation type such as deformation or change in surface appearance [1, 2]. This issue could be mitigated by the implementation of high-pressure freezing to achieve optimal preservation of internal structures in the foraminiferal cell during fixation.

For EDS elemental analyses, a flat surface of the sample is essential to produce good quality measurements and prevent the introduction of shadowing. This helps to avoid variable X-ray signal attenuations for each element due to local topographic variations on the µm scale [3]. In the case of a whole foraminiferal cell (up to 500 µm in its longer dimension for *B. spissa*), it is very challenging to obtain such a flat surface for the entire sample, even with the careful use of a diamond knife in the cryo-microtome.

Prior to SEM imaging and EDS analyses, a necessary step to expose foraminiferal ultrastructure is the sublimation of the overlying water ice that was deposited on the sample surface during transfer to the cryo-SEM chamber. The quantity of the overlying water and its distribution on the sample surface are variable, hence the sublimation time needed to expose the sample surface may also vary, in turn impacting the aspect of the water-rich cellular content. In this case, a longer sublimation time may enhance topographic contrast depending on the water distribution in the sample, and further result in a solute concentration through water removal [1].

The elemental distribution maps acquired with EDS represent the sample subsurface, because of the 3D interaction of the electron beam with the sample. Consequently, the X-ray signal obtained on EDS maps integrates a subsurface volume, rather than solely accounting for the sample surface. The interaction volume is dependent on the electron incident beam energy settings for acquisition, the sample composition, and the topography of the sample [4]. Therefore, the signal given by EDS is integrated over a depth varying from few hundreds of nm to few µm. For Foraminifera, this interaction volume was estimated to be ~ 1 µm in depth using Monte Carlo simulation [5]. Even though this depth is substantially smaller than the size of the organelles discussed in this study, the presence of a thin layer of another material (e.g., overlying water) covering the underlying cytoplasm content can lead to the measurement of an elemental signal (EDS) without a visible corresponding structure on SEM images. Additionally, this effect may hamper the determination of the elemental composition of structures having a size of a few µm, such as peroxisomes or mitochondria.

In this study, the high-resolution SEM image for entire individuals was composed of numerous SEM images (tiles), manually assembled post-acquisition. Concurrently, elemental distribution maps from EDS, having a much smaller resolution, were manually superimposed on the SEM high-resolution images. These image resolution differences and the manual procedures applied may have introduced lags in positions (estimated to be up to a 1−3 µm in this study), and sometimes made the exact alignment between EDS maps and SEM images difficult.

Despite these limitations, preventing for direct measurement of absolute elemental concentrations in this study, we successfully computed elemental ratios between organelle content and the cytosol, allowing for semi-quantification. Qualitative and semi-quantitative cryo-SEM-EDS approaches are important as they can confirm the soluble content within the cell, confirm the presence of previously hypothesised organelles (here acidocalcisomes), and aid in the identification of unknown structures (soluble or not) such as the vacuoles in the proloculus containing Ba granules, or the composite calcite/opaline test in *B. spissa* [6].

Finally, the use of reference standards may help to obtain quantitative measurements [3, 5]. For instance, calibration with standard solutions of known NO^3−^ concentrations, imaged during signal acquisition for the vacuoles, could help to quantify the vacuolar NO_3_^−^ concentrations more precisely. Additionally, recent developments in the challenging implementation of correlated cryo-SEM and cryo-Nano-SIMS imaging will help to ultimately determine the N fluxes and distribution in the cell [7].

*References:*

1. Liang J, Xiao X, Chou T-M, Libera M. Freezing and sublimation effects on cryo-SEM imaging and microanalysis. *Microscopy and Microanalysis* 2019; **25**: 1108–1109.

2. Al-Amoudi A, Studer D, Dubochet J. Cutting artefacts and cutting process in vitreous sections for cryo-electron microscopy. *Journal of Structural Biology* 2005; **150**: 109–121.

3. Newbury DE, Ritchie NWM. Is Scanning Electron Microscopy/Energy Dispersive X-ray Spectrometry (SEM/EDS) Quantitative? *Scanning* 2013; **35**: 141–168.

4. Goldstein JI, Newbury DE, Michael JR, Ritchie NWM, Scott JHJ, Joy DC. Electron Beam—Specimen Interactions: Interaction Volume. *Scanning Electron Microscopy and X-Ray Microanalysis*. 2018. Springer New York, New York, NY, pp 1–14.

5. Khalifa GM, Kahil K, Erez J, Kaplan Ashiri I, Shimoni E, Pinkas I, et al. Characterization of unusual MgCa particles involved in the formation of foraminifera shells using a novel quantitative cryo SEM/EDS protocol. *Acta Biomaterialia* 2018; **77**: 342–351.

6. Richirt J, Okada S, Ishitani Y, Uematsu K, Tame A, Oda K, et al. Composite calcite and opal test in Foraminifera (Rhizaria). *Biogeosciences* 2024; **21**: 3271–3288.

7. Meibom A, Plane F, Cheng T, Grandjean G, Haldimann O, Escrig S, et al. Correlated cryo-SEM and CryoNanoSIMS imaging of biological tissue. *BMC Biology* 2023; **21**: 126.


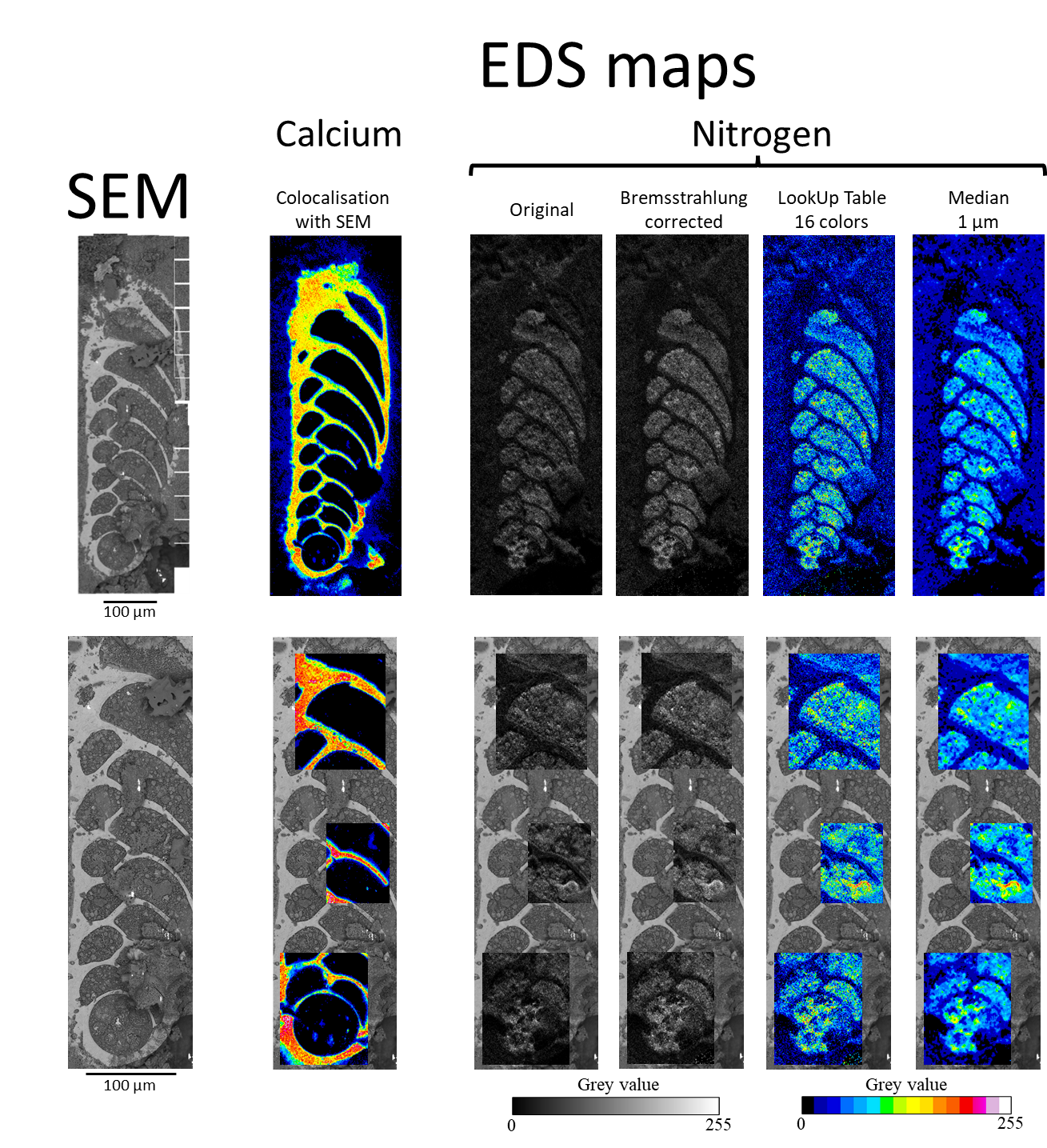


*Supplementary Figure 1: Example of SEM-EDS colocalisation using EDS-Ca distribution and image treatment performed on N-EDS maps from original EDS map to finale analysed distribution map for an individual incubated in condition oxic and 40 µM of nitrate. The top part represents a complete individual mapped, and the bottom part shows the regions of interest (ROI) for the same individual.*


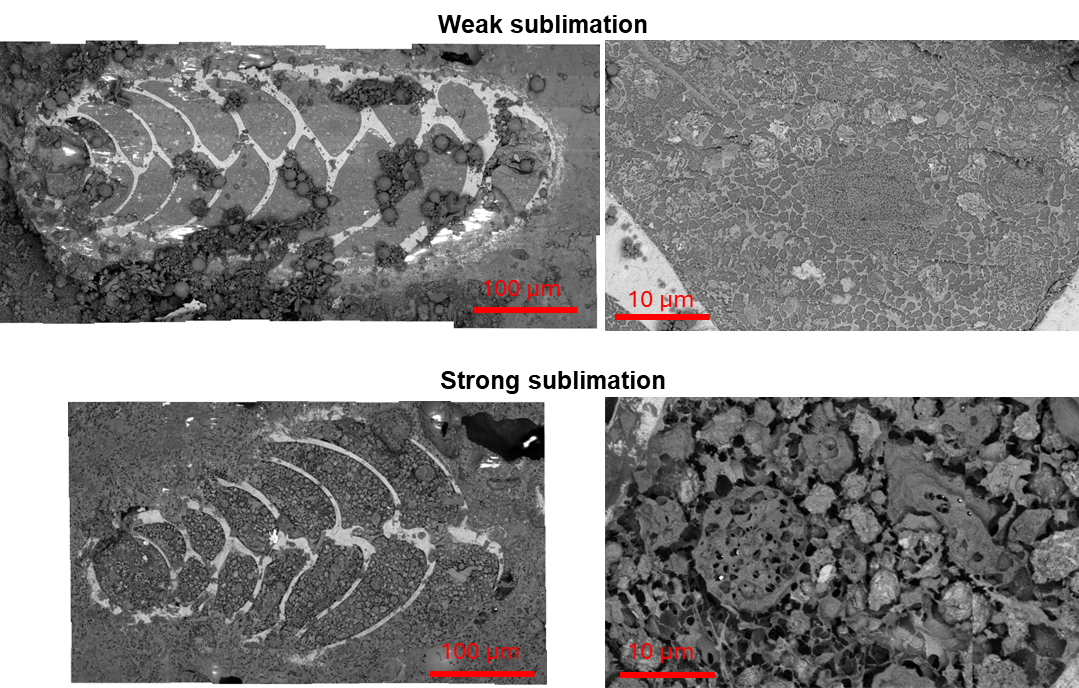


*Supplementary Figure 2: Example of the sample surface aspect after sublimation for two* B. spissa *specimens. While the top specimen (anoxic40-D) shows a relatively weak removal of water, the bottom individual (anoxic40-A) shows a strong removal of water. For both specimens, sublimation duration was similar.*

*
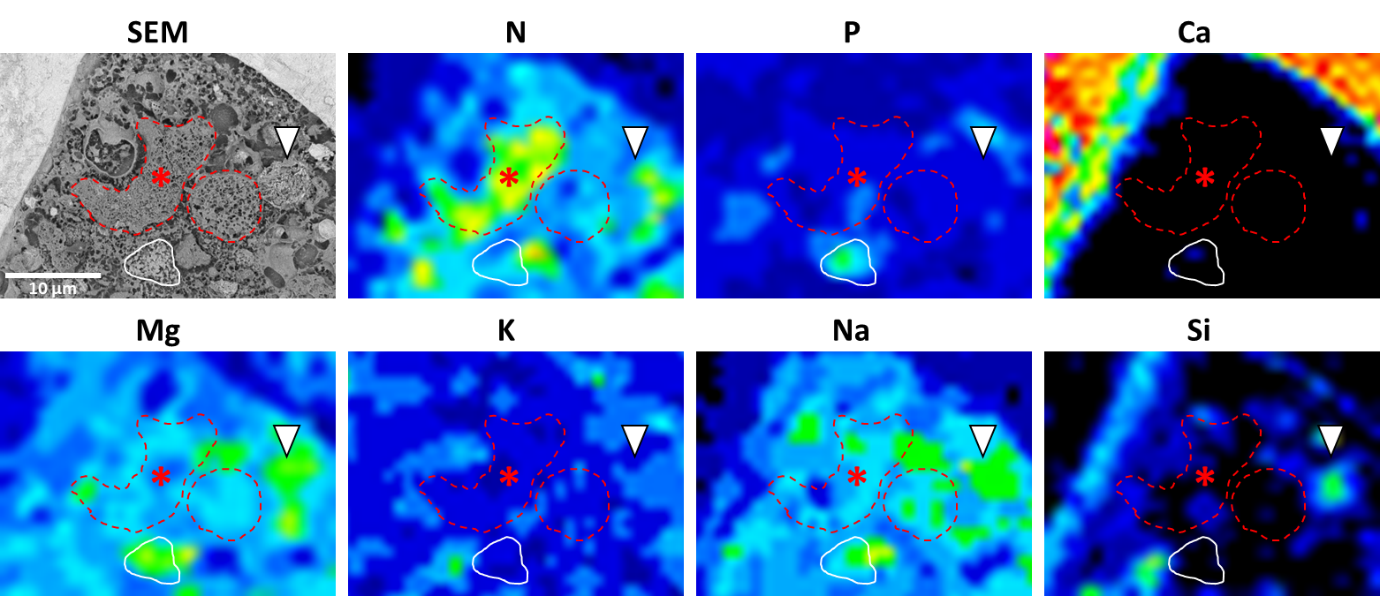
*

*Supplementary Figure 3: Example of cryo-SEM image and EDS maps for individual oxic40-A (same as in figures 4, 5 and 6 of the main manuscript) showing a N-enriched vacuole (red dotted line with an asterisk) and a N-empty vacuole (red dotted lines without asterisk). The white outline indicates an acidocalcisome, enriched in P, Mg and Ca. Finally, the white arrowhead indicates a degradation vacuole, rich in Si (material from sedimentary origin).*

*Supplementary Figure 4: Histogram showing the enrichment factor regarding the incubation condition. All vacuoles considered (N-enriched and not N-enriched).*

*
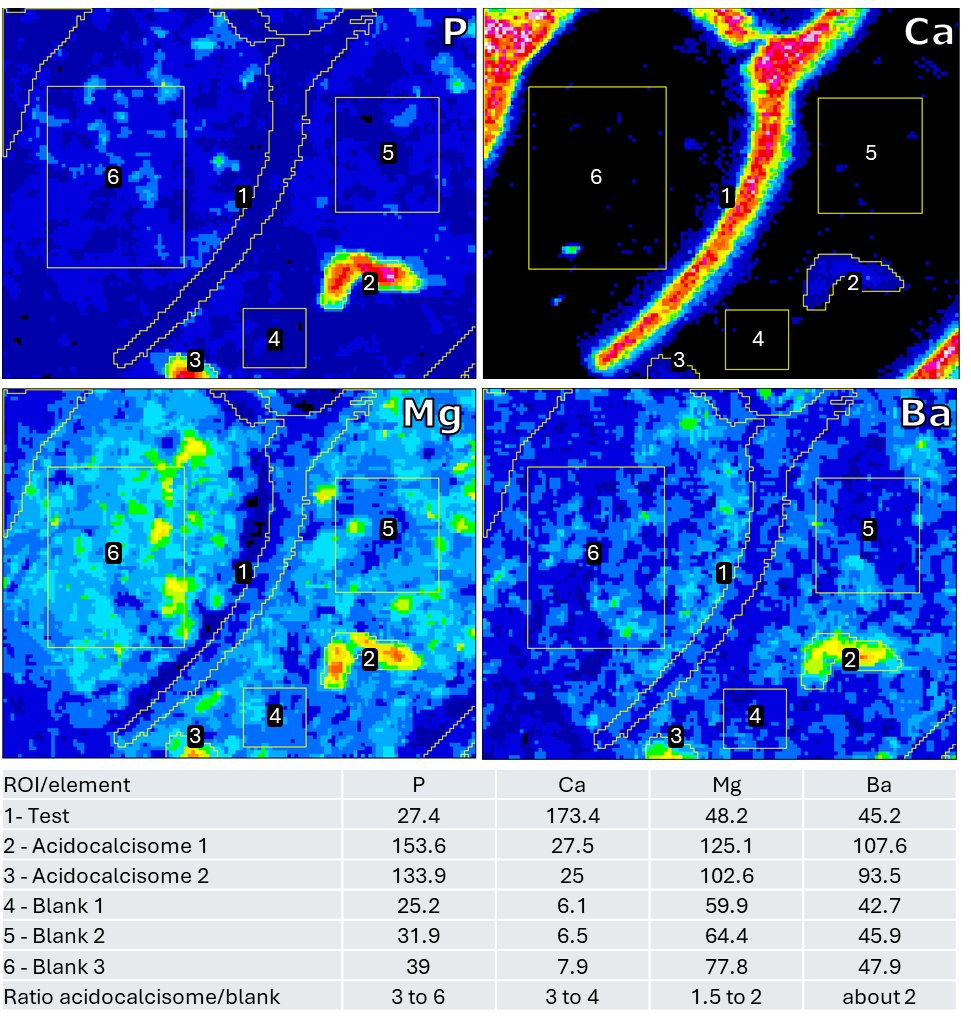
*

*Supplementary Figure 5: EDS maps showing how the elemental enrichment factors for P, Ca, Mg and Ba in acidocalcisomes were estimated for the same individual than in figures 4, 5 & 6 in the main text (oxic40-A).*

*
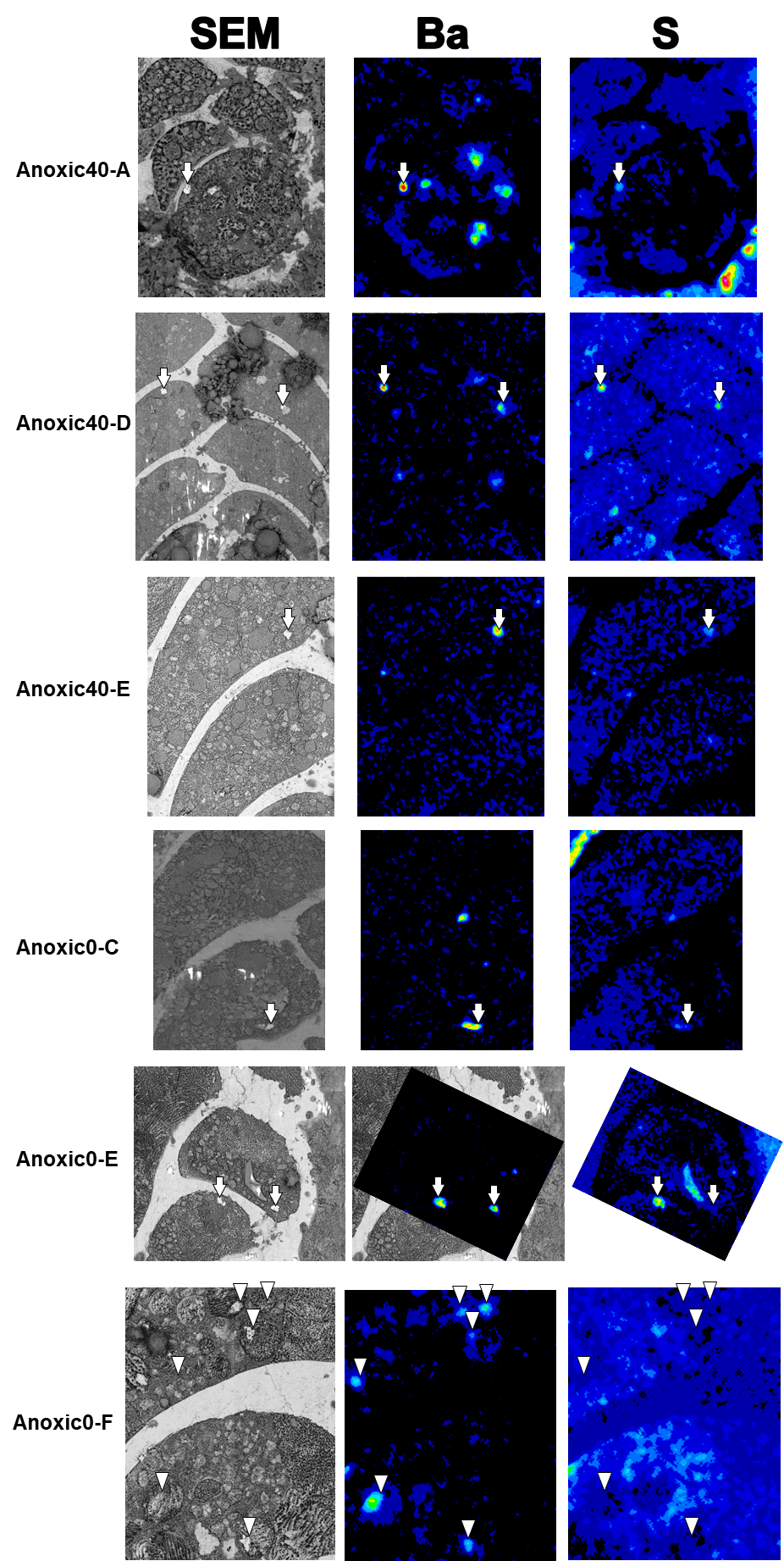
*

*Supplementary Figure 6: SEM image, Ba-EDS and S-EDS distributions colocalised with the corresponding SEM image. White arrows indicate the presence particles not encompassed in a vacuole and being rich in Ba associated with S, localised in the proloculus for individual anoxic40-A and outside the proloculus for individuals anoxic0-C, anoxic40-D, anoxic40-E and anoxic0-E. White arrowheads on SEM and EDS maps for individual Anoxic0-F indicate Ba-rich particles encompassed in vacuoles and not enriched in S, outside the proloculus.*


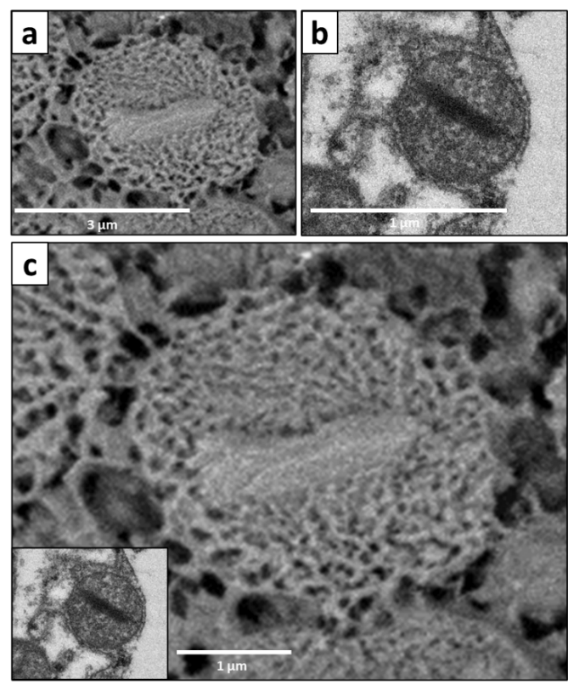


*Supplementary Figure 7: Cryo-SEM (a) and TEM (b) image of putative peroxisomes in* B. spissa*, exhibiting the typical catalase core (Bernhard & Bowser, 2008). Note the scale difference, SEM images being three times magnified compared to TEM image. (c) Same images than a and b, scaled to emphasise size difference.*

*
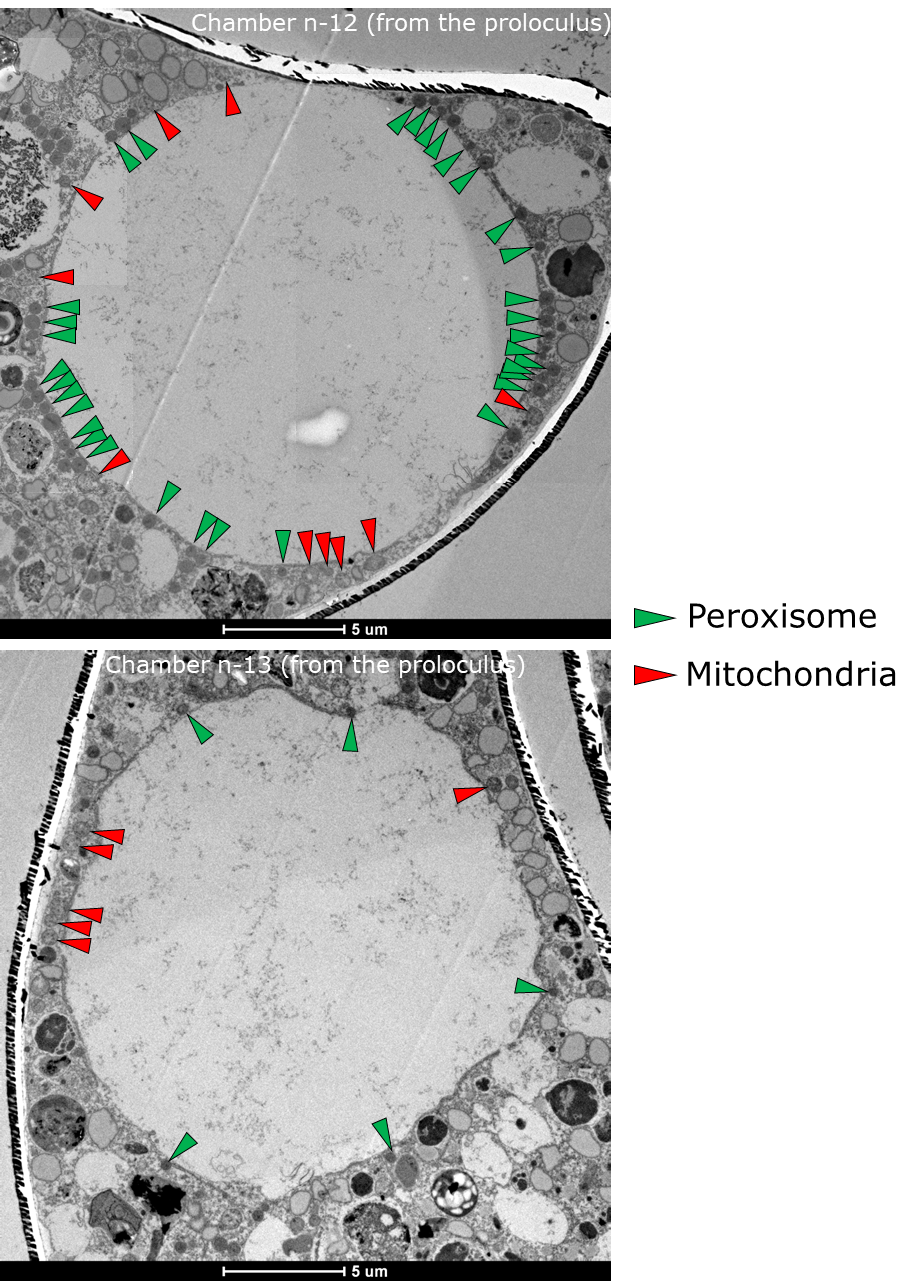
*

*Supplementary Figure 8: TEM images of* B. spissa *showing two vacuoles located in two consecutive chambers (n-12 and n-13) of the same individual. Peroxisomes are indicated by green arrowheads and mitochondria are indicated by red arrowheads. Note the important difference in mitochondria and peroxisome density at the edge of each vacuole.*

*
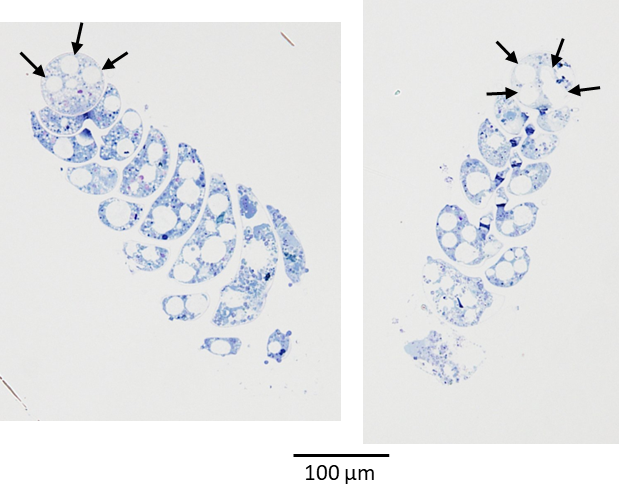
*

*Supplementary Figure 9: Semi-thin sections of* B. spissa *observed under optic microscope. Vacuoles located in the proloculus are indicated with black arrows.*

*Supplementary Table 1: for all the cryo-fixed individuals, sampling periods, individual ID, origin of specimens, oxygen and nitrate concentrations, sublimation state, presence or absence of decalcification marks, collapsed cellular content, broken test pieces, SEM charging regions and potential sample surface covering by unwanted material, visible cytoplasm area visible on SEM image, general quality state of SEM images based on a global assessment, general quality of EDS maps and visibility of the proloculus on SEM images. Individuals in* ***bold text*** *were considered of too poor quality and were discarded from further analysis.*

| **Sampling period** | **Individual ID** | **Individual origin** | **Oxygen (oxic/anoxic)** | **Nitrate concentration (µM)** | **Sublimation state** | **Decalcification marks** | **Collapsed cellular content** | **Broken test pieces** | **SEM charging** | **Surface covered** | **Visible cytoplasm area (mm²)** | **General quality state of sample (SEM)** | **General quality state of EDS acquisition** | **Proloculus visible on SEM** |
| --- | --- | --- | --- | --- | --- | --- | --- | --- | --- | --- | --- | --- | --- | --- |
| October 2022 | field-A | Field | na | na | GOOD | NO | NO | NO | NO | NO | 0.35 | GOOD | GOOD | NO |
| October 2022 | **field-B** | Field | na | na | STRONG | NO | YES | YES | YES | NO | na | **BAD** | GOOD | YES |
| May 2023 | field-C | Field | na | na | WEAK | NO | YES (proloculus only) | NO | YES (weak) | NO | 0.40 | GOOD | GOOD | YES |
| October 2022 | oxic0-A | Experiment 1 | Oxic | 0 | GOOD | NO | NO | NO | NO | NO | 0.24 | GOOD | GOOD | YES |
| October 2022 | oxic0-B | Experiment 1 | Oxic | 0 | GOOD | NO | NO | NO | NO | NO | 0.38 | GOOD | GOOD | NO |
| October 2022 | oxic0-C | Experiment 1 | Oxic | 0 | GOOD | NO | YES (only 1 chamber) | NO | NO | YES (glue) | 0.30 | GOOD | GOOD | NO |
| October 2022 | oxic40-A | Experiment 1 | Oxic | 40 | GOOD | NO | NO | NO | NO | YES (glue, partly) | 0.40 | GOOD | GOOD | YES |
| October 2022 | **oxic40-B** | Experiment 1 | Oxic | 40 | GOOD | NO | NO | NO | NO | YES | na | **BAD** | GOOD | NO |
| October 2022 | oxic40-C | Experiment 1 | Oxic | 40 | WEAK | NO | NO | NO | YES (weak) | NO | 0.31 | GOOD | GOOD | NO |
| October 2022 | **anoxic0-A** | Experiment 1 | Anoxic | 0 | STRONG | YES | YES (almost completely) | NO | YES (weak) | NO | na | **BAD** | GOOD | YES |
| October 2022 | **anoxic0-B** | Experiment 1 | Anoxic | 0 | WEAK | YES | YES | NO | YES (weak) | NO | na | **BAD** | GOOD | NO |
| October 2022 | anoxic0-C | Experiment 1 | Anoxic | 0 | GOOD | YES | NO | NO | YES (weak) | NO | 0.24 | GOOD | GOOD | NO |
| October 2022 | **anoxic0-D** | Experiment 1 | Anoxic | 0 | WEAK | YES | YES | NO | NO | YES (glue) | na | **BAD** | GOOD | NO |
| May 2023 | anoxic0-E | Experiment 2 | Anoxic | 0 | GOOD | NO | NO | NO | NO | NO | 0.22 | GOOD | GOOD | NO |
| May 2023 | anoxic0-F | Experiment 2 | Anoxic | 0 | GOOD | NO | NO | NO | NO | NO | 0.33 | GOOD | GOOD | YES |
| May 2023 | **anoxic0-G** | Experiment 2 | Anoxic | 0 | WEAK | NO | NO | NO | NO | YES (water) | 0.21 | GOOD | **BAD** | YES |
| October 2022 | anoxic40-A | Experiment 1 | Anoxic | 40 | GOOD | YES | NO | NO | NO | NO | 0.21 | GOOD | GOOD | YES |
| October 2022 | anoxic40-B | Experiment 1 | Anoxic | 40 | GOOD | YES | NO | NO | YES (weak) | NO | 0.16 | GOOD | GOOD | NO |
| October 2022 | anoxic40-C | Experiment 1 | Anoxic | 40 | GOOD | YES | NO | NO | YES (weak) | NO | 0.21 | GOOD | GOOD | NO |
| May 2023 | anoxic40-D | Experiment 2 | Anoxic | 40 | WEAK | NO | YES (few early chambers) | NO | NO | YES (water) | 0.38 | GOOD | GOOD | YES |
| May 2023 | anoxic40-E | Experiment 2 | Anoxic | 40 | GOOD | NO | YES (proloculus only) | NO | NO | NO | 0.48 | GOOD | GOOD | YES |
| May 2023 | anoxic40-F | Experiment 2 | Anoxic | 40 | GOOD | NO | YES (partly) | NO | NO | YES (glue) | 0.26 | GOOD | GOOD | YES |

*Supplementary Table 2: Individual ID, origin, oxygen and nitrate concentrations in experimental setup, number and size range of identified vacuole(s) on SEM, number of N-enriched and non-N-enriched vacuole(s), percentage of N-enriched vacuole(s) over the total number of identified vacuole(s) and N-enrichment factor of vacuole(s) considered N-enriched compared to cytoplasm.*

| **Individual ID** | **Individual origin** | **Oxygen (oxic/anoxic)** | **Nitrate concentration in µM** | **Number of identified vacuoles on SEM** | **Size range of vacuoles (SEM images)** | **Number of N-enriched vacuole(s)** | **number of non-N-enriched vacuole(s)** | **Percentage of N-enriched vacuole(s)** | **N-enrichment factor in N-enriched vacuoles, average ± sd** |  |
| --- | --- | --- | --- | --- | --- | --- | --- | --- | --- | --- |
| field-A | Field | na | na | 6 | 8-35 µm | 6 | 0 | 100 | 2.6 ± 0.6 |  |
| field-C | Field | na | na | 0 | na | na | na | na | na |  |
| oxic0-A | Experiment 1 | Oxic | 0 | 12 | 7-31 µm | 9 | 3 | 75 | 1.7 ± 0.2 |  |
| oxic0-B | Experiment 1 | Oxic | 0 | 24 | 5-29 µm | 21 | 3 | 88 | 2.0 ± 0.3 |  |
| oxic0-C | Experiment 1 | Oxic | 0 | 8 | 8-22 µm | 8 | 0 | 100 | 1.9 ± 0.2 |  |
| oxic40-A | Experiment 1 | Oxic | 40 | 20 | 6-35 µm | 18 | 2 | 90 | 1.7 ± 0.1 |  |
| oxic40-C | Experiment 1 | Oxic | 40 | 0 | na | na | na | na | na |  |
| anoxic0-C | Experiment 1 | Anoxic | 0 | 14 | 5-29 µm | 13 | 1 | 93 | 2.3 ± 0.5 |  |
| anoxic0-E | | Experiment 2 | Anoxic | 0 | 7 | 5-14 µm | 7 | 0 | 100 | 2.3 ± 0.2 |
| anoxic0-F | | Experiment 2 | Anoxic | 0 | 0 | na | na | na | na | na |
| anoxic40-A | Experiment 1 | Anoxic | 40 | 24 | 7-37 µm | 19 | 5 | 79 | 2.0 ± 0.3 |  |
| anoxic40-B | Experiment 1 | Anoxic | 40 | 5 | 8-17 µm | 5 | 0 | 100 | 2.0 ± 0.3 |  |
| anoxic40-C | Experiment 1 | Anoxic | 40 | 10 | 7-14 µm | 10 | 0 | 100 | 2.4 ± 0.5 |  |
| anoxic40-D | Experiment 2 | Anoxic | 40 | 19 | 3-15 µm | 12 | 7 | 63 | 1.6 ± 0.3 |  |
| anoxic40-E | Experiment 2 | Anoxic | 40 | 74 | 4-33 µm | 27 | 47 | 36 | 1.8 ± 0.3 |  |
| anoxic40-F | Experiment 2 | Anoxic | 40 | 10 | 6-23 µm | 6 | 4 | 54 | 1.4 ± 0.4 |  |

*Supplementary Table 3: Individual ID, origin, oxygen and nitrate concentrations in experimental setup, number and size range of identified acidocalcisome(s) on SEM, number of P-enriched and non-P-enriched vacuole(s), percentage of P-enriched acidocalcisome(s) over the total number of identified acidocalcisome(s), P-enrichment factor of acidocalcisome(s) compared to cytoplasm and other element(s) occurring in acidocalcisome(s) with their respective enrichment factors (between brackets).*

| **Individual ID** | **Individual origin** | **Oxygen (oxic/anoxic)** | **Nitrate concentration in µM** | **Number of identified acidocalcisomes** | **Size range of acidocalcisomes** | **Number of P-enriched acidocalcisome(s)** | **Number of non P-enriched acidocalcisome(s)** | **Percentage of P-enriched acidocalcisome(s)** | **P-enrichment factor in acidocalcisome(s)** | **Presence of other elements in acidocalcisome(s) (enrichment factor)** |
| --- | --- | --- | --- | --- | --- | --- | --- | --- | --- | --- |
| field-A | Field | na | na | 8 | 4-13 µm | 5 | 3 | 63 | 3-4 times | Ca (3 times)  Mg (1.5-2 times)  Ba (4 times) |
| field-C | Field | na | na | 4 | 4-9 µm | 4 | 0 | 100 | 3-4 times | Ca (2-3 times) |
| oxic0-A | Experiment 1 | Oxic | 0 | 0 | na | na | na | na | na | na |
| oxic0-B | Experiment 1 | Oxic | 0 | 7 | 3-8 µm | 7 | 0 | 100 | 3 times | Ca (3 times) |
| oxic0-C | Experiment 1 | Oxic | 0 | 0 | na | na | na | na | na | na |
| oxic40-A | Experiment 1 | Oxic | 40 | 15 | 3-19 µm | 14 | 1 | 93 | 3-6 times | Ca (2-4 times)  Mg (2 times)  Ba (2-3 times) |
| oxic40-C | Experiment 1 | Oxic | 40 | 7 | 4-10 µm | 7 | 0 | 100 | 2-5 times | Ca (2-5 times)  Mg (1.5-2 times) |
| anoxic0-C | Experiment 1 | Anoxic | 0 | 0 | na | na | na | na | na | na |
| anoxic0-E | Experiment 2 | Anoxic | 0 | 0 | na | na | na | na | na | na |
| anoxic0-F | Experiment 2 | Anoxic | 0 | 0 | na | na | na | na | na | na |
| anoxic40-A | Experiment 1 | Anoxic | 40 | 3 | 8 µm | 2 | 1 | 67 | 4 times | Ca (2 times)  Mg (1.5 times) |
| anoxic40-B | Experiment 1 | Anoxic | 40 | 0 | na | na | na | na | na | na |
| anoxic40-C | Experiment 1 | Anoxic | 40 | 2 | 5-6 µm | 1 | 1 | 50 | 2-4 times | na |
| anoxic40-D | Experiment 2 | Anoxic | 40 | 9 | 4-9 µm | 8 | 1 | 89 | 4-7 times | Ca (5-6 times)  Mg (1.5-2 times)  Ba (2-4 times) |
| anoxic40-E | Experiment 2 | Anoxic | 40 | 25 | 3-11 µm | 10 | 15 | 40 | 3-4 times | Ca (3-6 times)  Mg (1.5-2 times)  Ba (2-5 times) |
| anoxic40-F | Experiment 2 | Anoxic | 40 | 4 | 5-10 µm | 4 | 4 | 100 | 3 times | Ca (2 times)  Mg (2 times)  Ba (2 times) |

*Supplementary Table 4: Individual ID, origin, oxygen and nitrate concentrations in experimental setup, number and size range of identified vacuole(s) in the proloculus on SEM, number of K-enriched and non-K-enriched vacuole(s) in the proloculus, number of identified grape-like structures located inside the vacuoles in the proloculus, number of Ba-enriched grape-like structures located inside the vacuoles in the proloculus and their size range.*

| **Individual ID** | **Individual origin** | **Oxygen (oxic/anoxic)** | **Nitrate concentration in µM** | **Number of identified vacuole(s) in the proloculus** | **Size range of the vacuole(s) in the proloculus** | **Number of K-enriched vacuole(s) in the proloculus** | **Number of Na-poor vacuole(s) in the proloculus** | **Number of identified granular structure(s) in the proloculus** | **Number of Ba-enriched granular structure(s) in the proloculus** | **Size range of Ba-enriched grape-like structure(s) in the proloculus** |
| --- | --- | --- | --- | --- | --- | --- | --- | --- | --- | --- |
| field-C | Field | na | na | 3 | 15-33 µm | 2 | 3 | 2 | 2 | 4-6 µm |
| oxic0-A | Experiment 1 | Oxic | 0 | 0 | na | 0 | 0 | 0 | 0 | na |
| oxic40-A | Experiment 1 | Oxic | 40 | 7 | 17-25 µm | 7 | 7 | 4 | 3 | 4-9 µm |
| anoxic0-F | Experiment 2 | Anoxic | 0 | 7 | 9-25 µm | 3 | 3 | 3 | 2 | 3-11 µm |
| anoxic40-A | Experiment 1 | Anoxic | 40 | 6 | 18-22 µm | 6 | 6 | 4 | 4 | 4-8 µm |
| anoxic40-D | Experiment 2 | Anoxic | 40 | 4 | 16-19 µm | 3 | 4 | 0 | 0 | na |
| anoxic40-E | Experiment 2 | Anoxic | 40 | 4 | 15-18 µm | 2 | 2 | 0 | 0 | na |
| anoxic40-F | Experiment 2 | Anoxic | 40 | 7 | 16-25 µm | 6 | 6 | 6 | 6 | 4-5 µm |
