# Supplementary material for: Different Elemental Compositions and Potential Functions of Vacuoles in Bolivina spissa (Foraminifera, Rhizaria) Based on Cryo‐SEM‐EDS Analyses
Source: J Eukaryot Microbiol. 2025 Aug 31;72(5):e70044. doi: 10.1111/jeu.70044 (PMC12399780; doi:10.1111/jeu.70044)
Supplement: Supplementary file 2 — Data S2: jeu70044‐sup‐0002‐DataS2.pdf. [file JEU-72-e70044-s002.pdf]

field-A

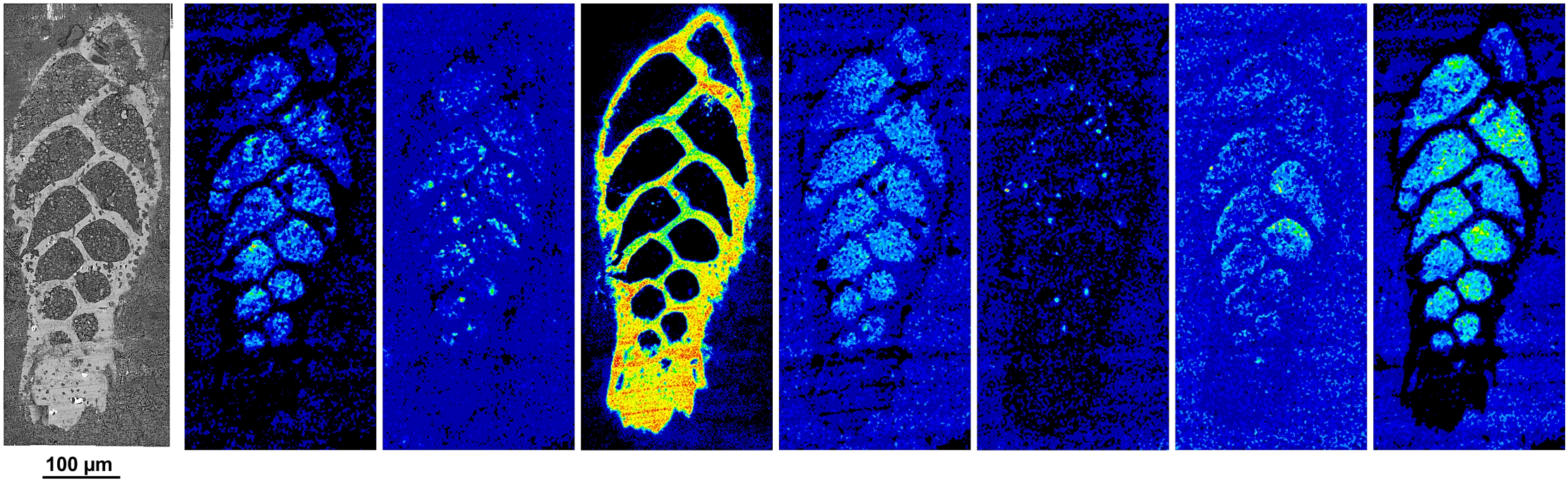

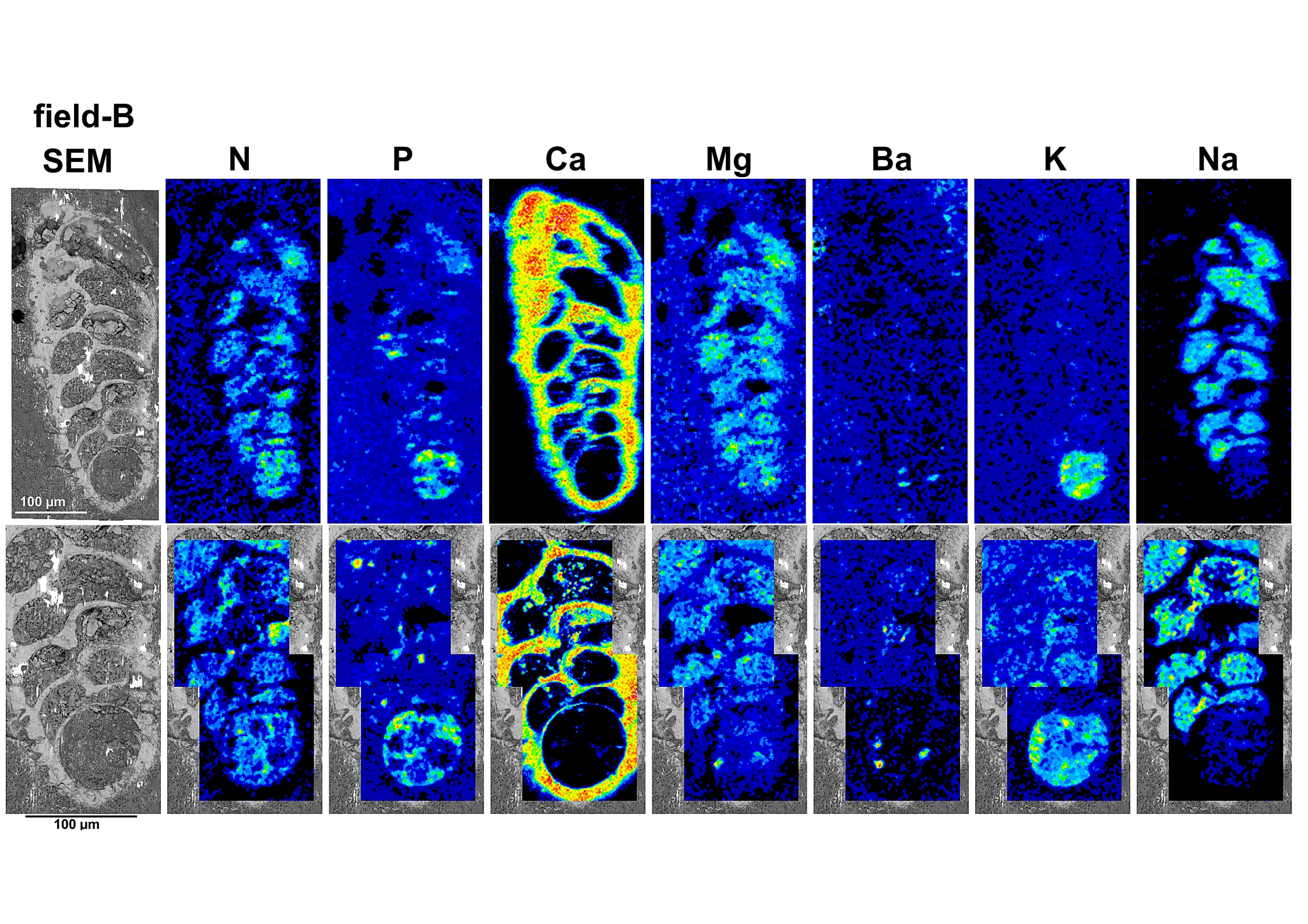
field-B
SEM
N
P
Ca
Mg
Ba
K
Na
100 μm
100 μm

field-C

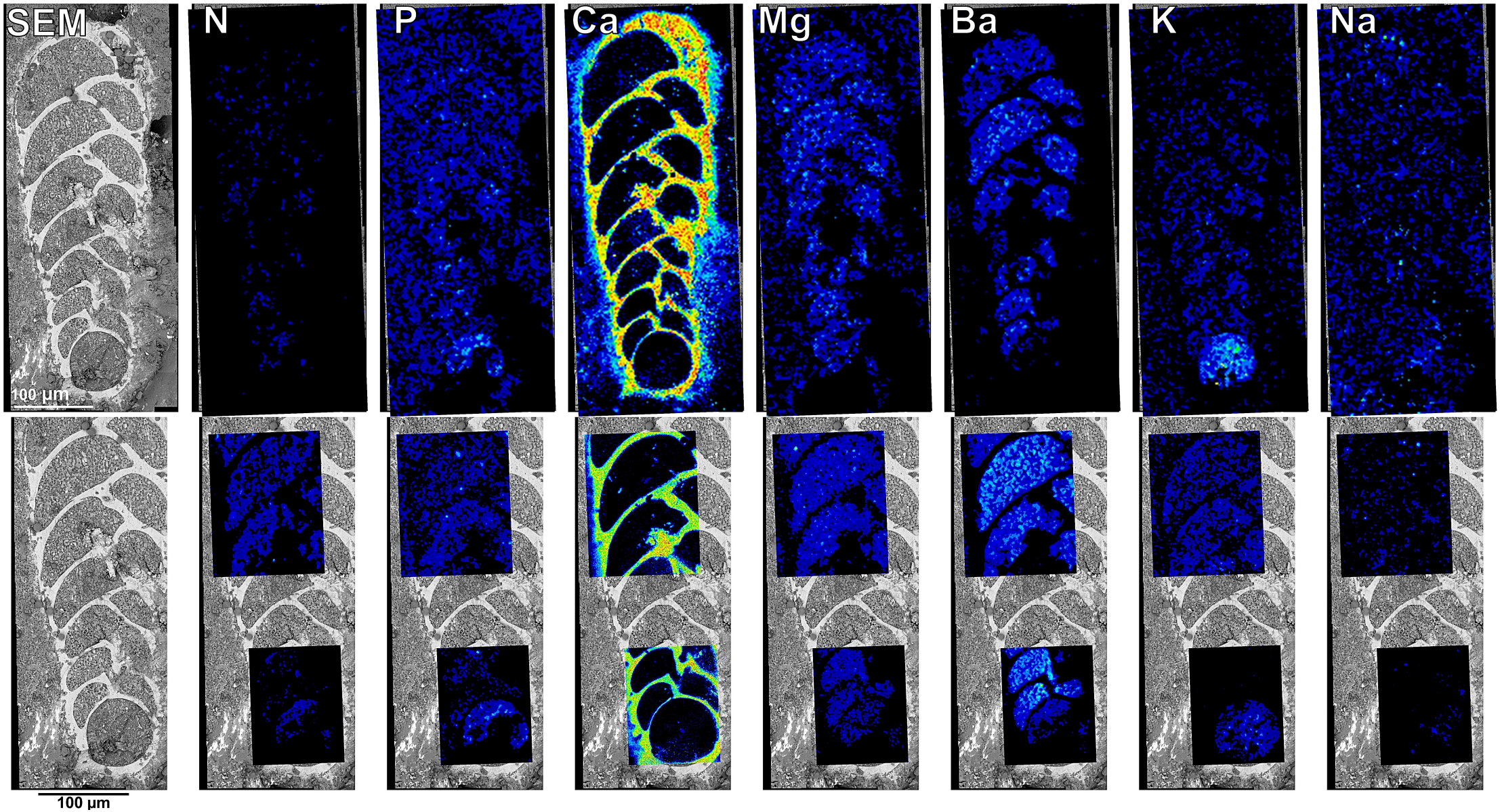

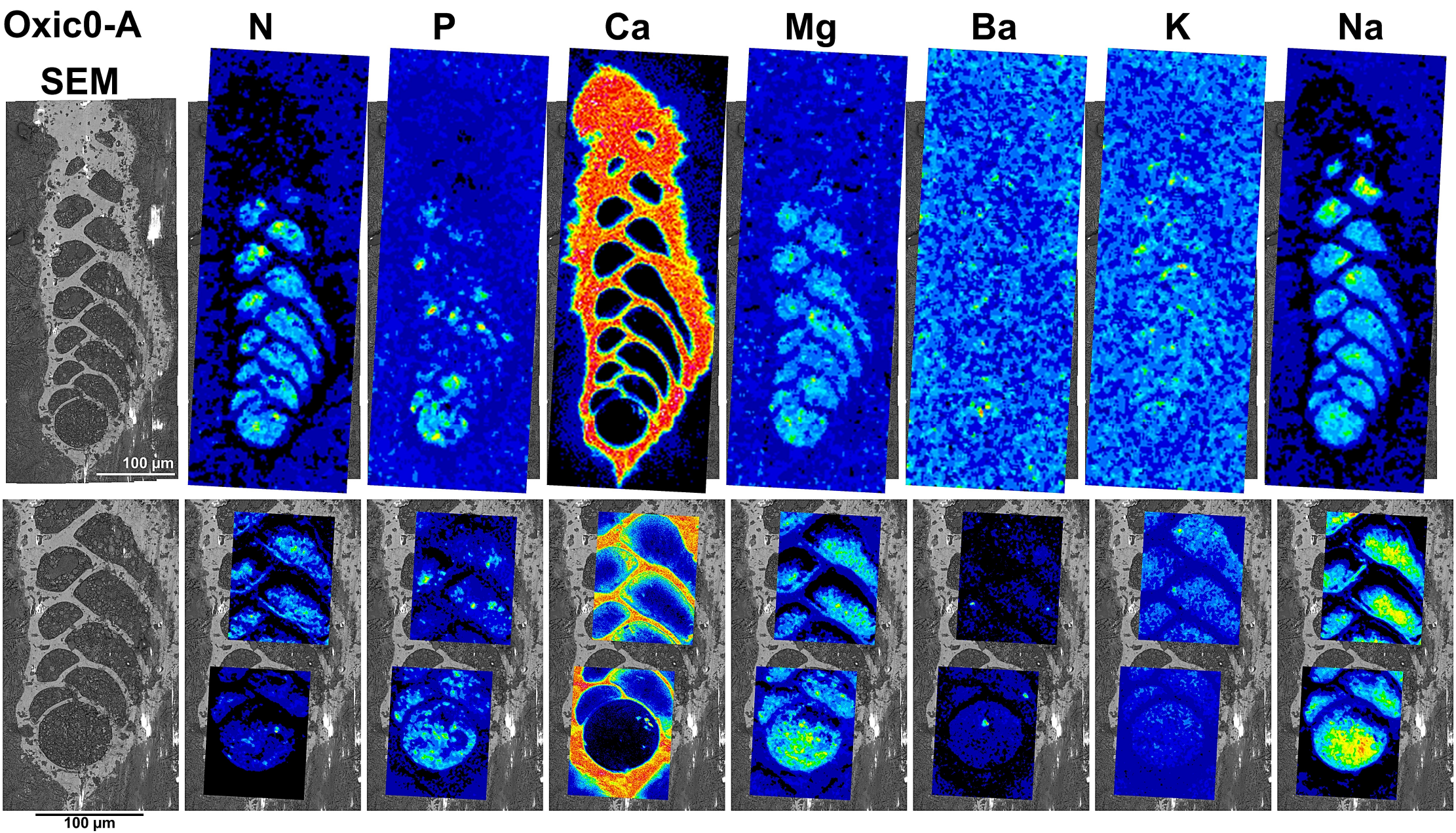
Oxic0-A
SEM
N
P
Ca
Mg
Ba
K
Na
100 µm
100 µm

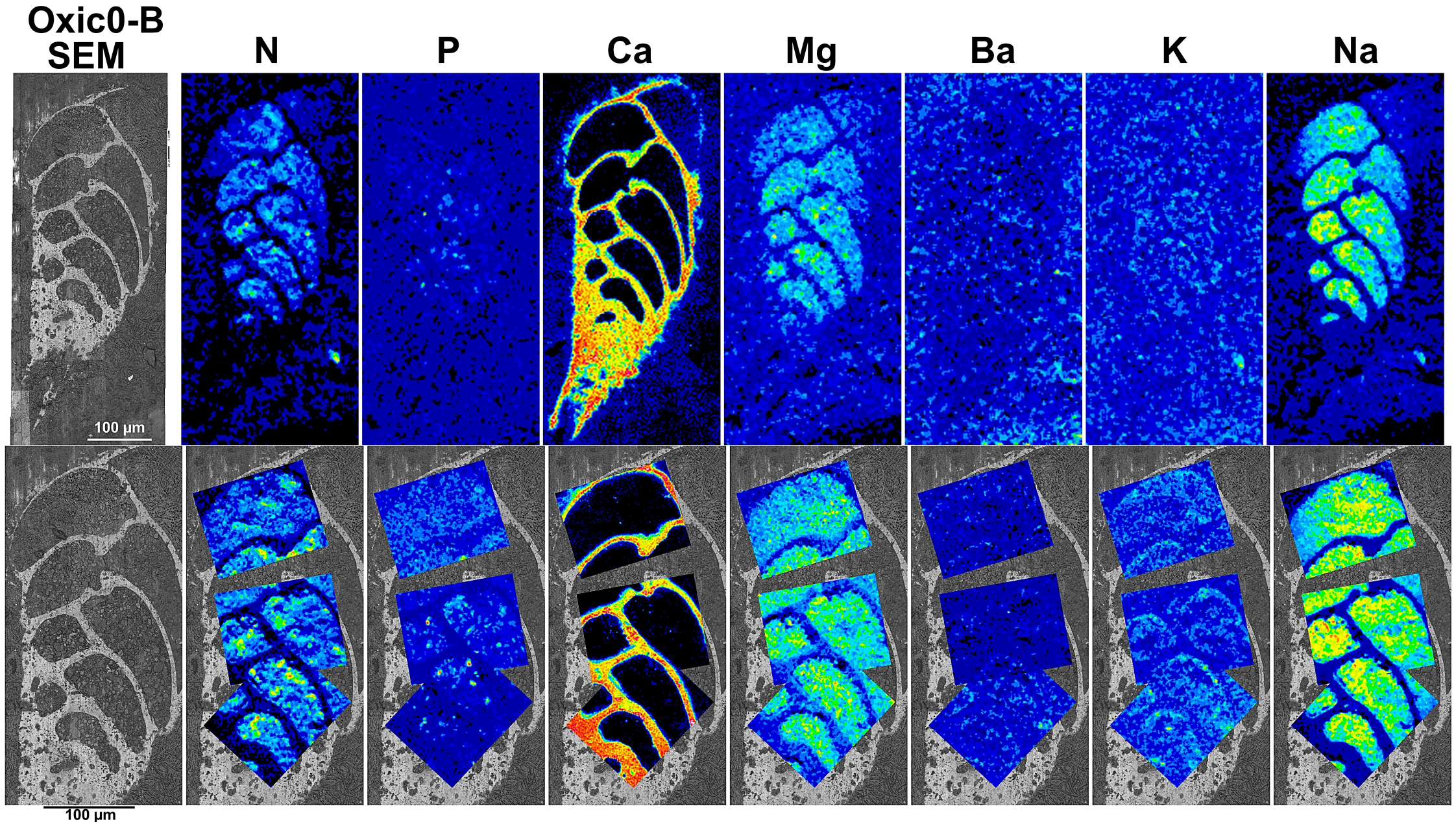
Oxic0-B
SEM
N
P
Ca
Mg
Ba
K
Na
100 µm
100 µm

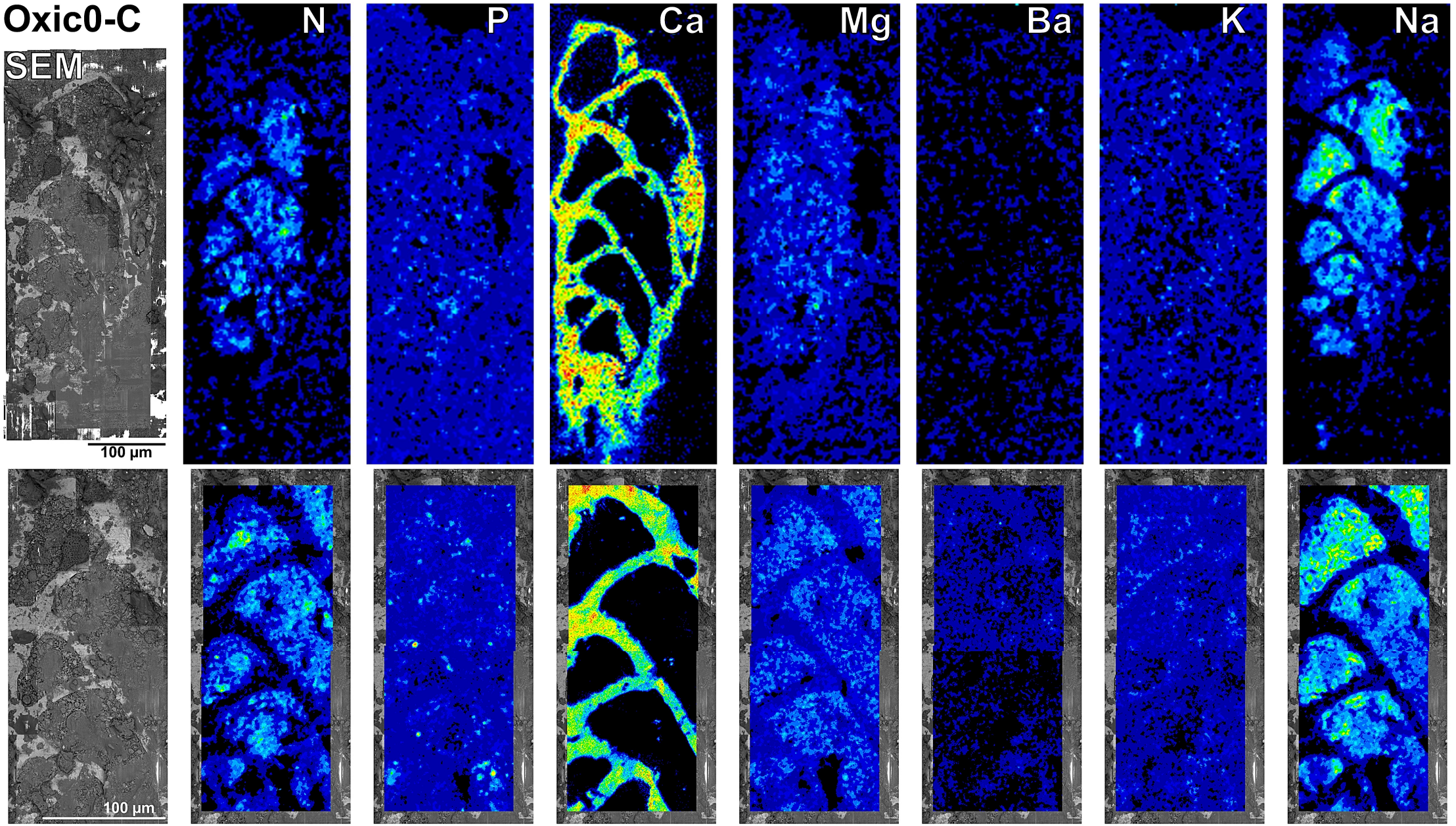
Oxic0-C
SEM
100 µm
N
P
Ca
Mg
Ba
K
Na
100 µm

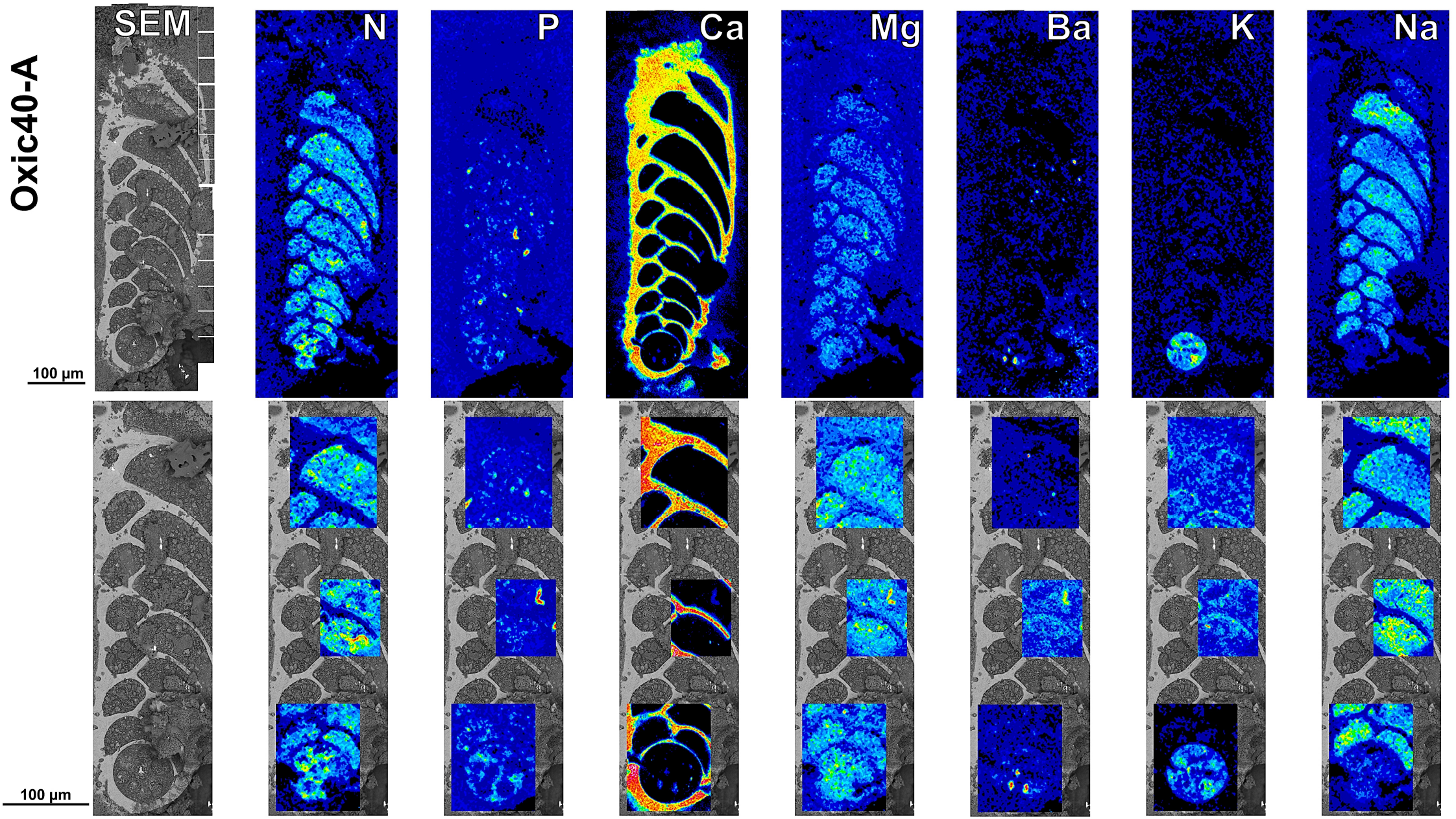
Oxic40-A
SEM
N
P
Ca
Mg
Ba
K
Na
100 µm
100 µm

Oxic40-B

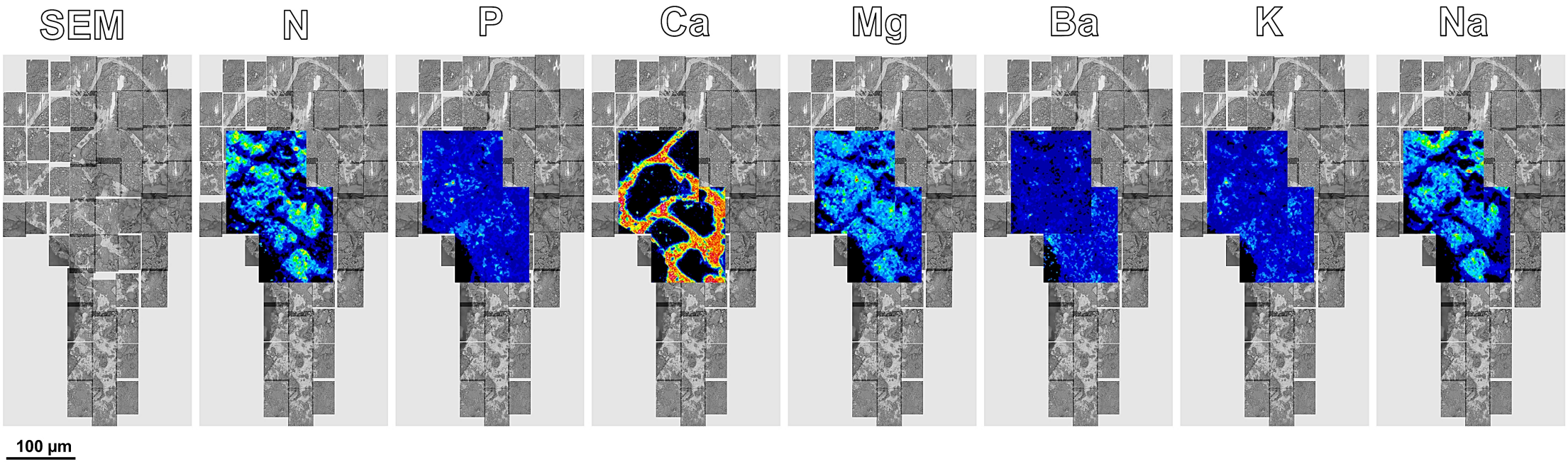

Oxic40-C

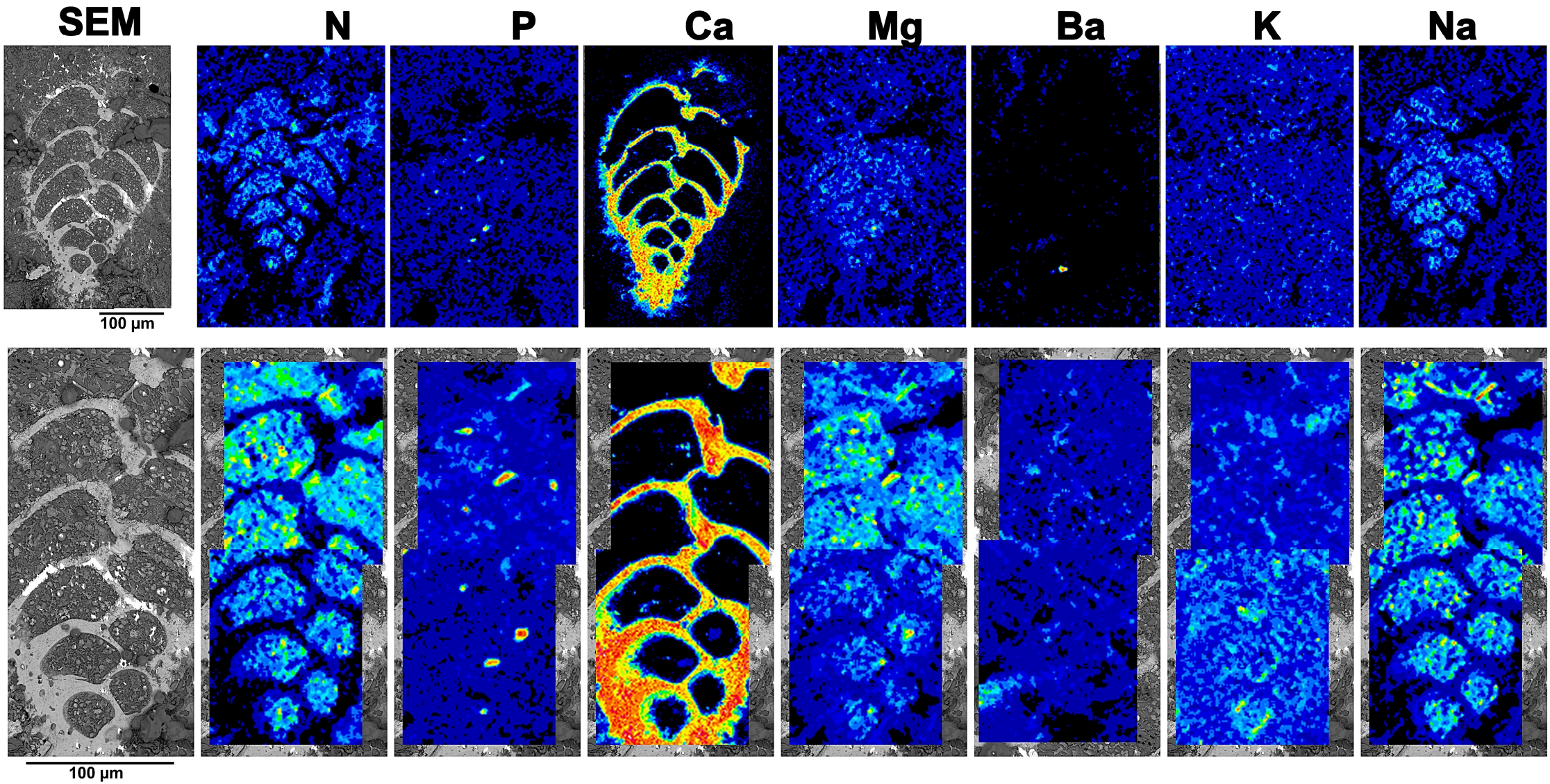

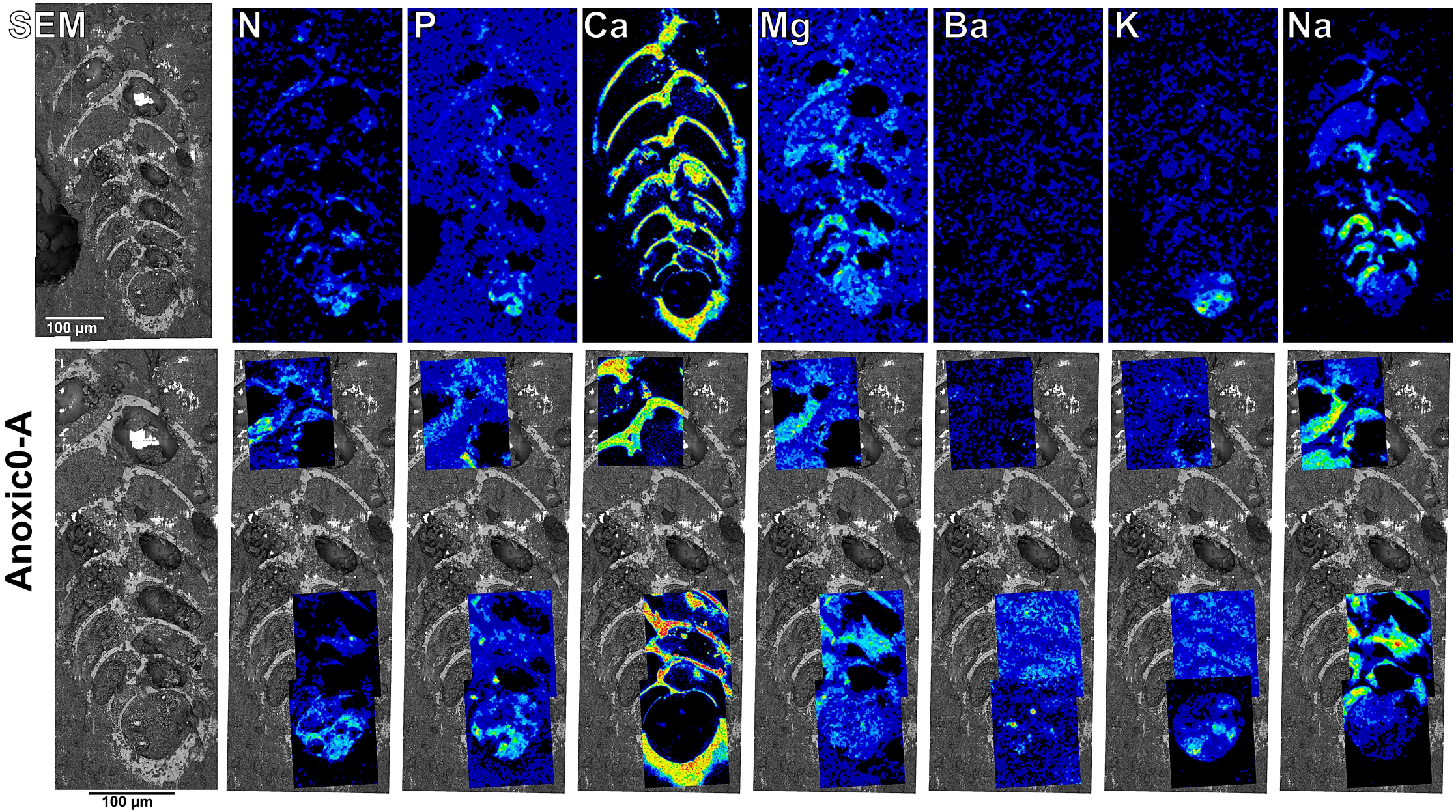
SEM
N
P
Ca
Mg
Ba
K
Na
100 µm
Anoxic0-A
100 µm

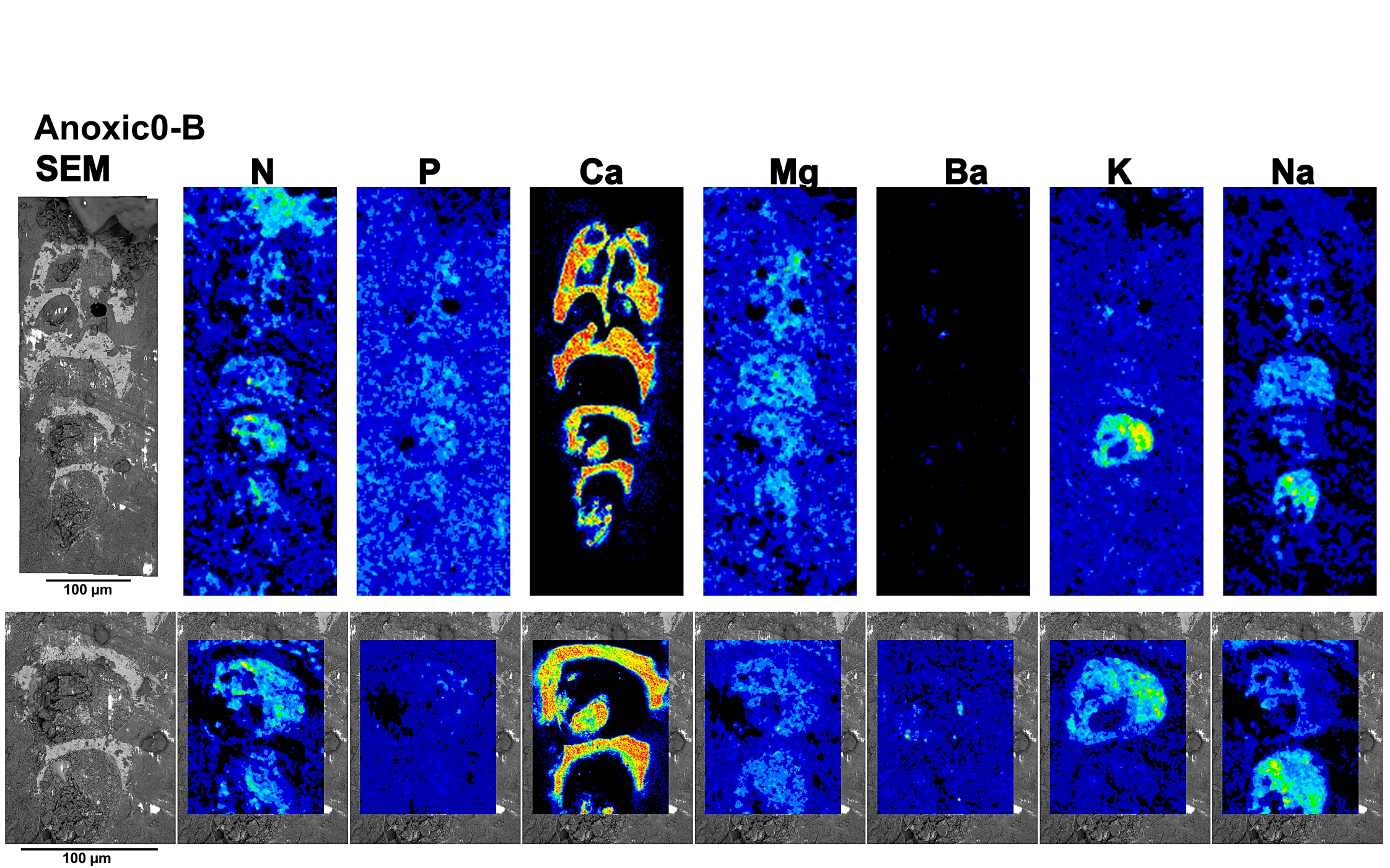
Anoxic0-B
SEM
N
P
Ca
Mg
Ba
K
Na
100 µm
100 µm

Anoxic0-C

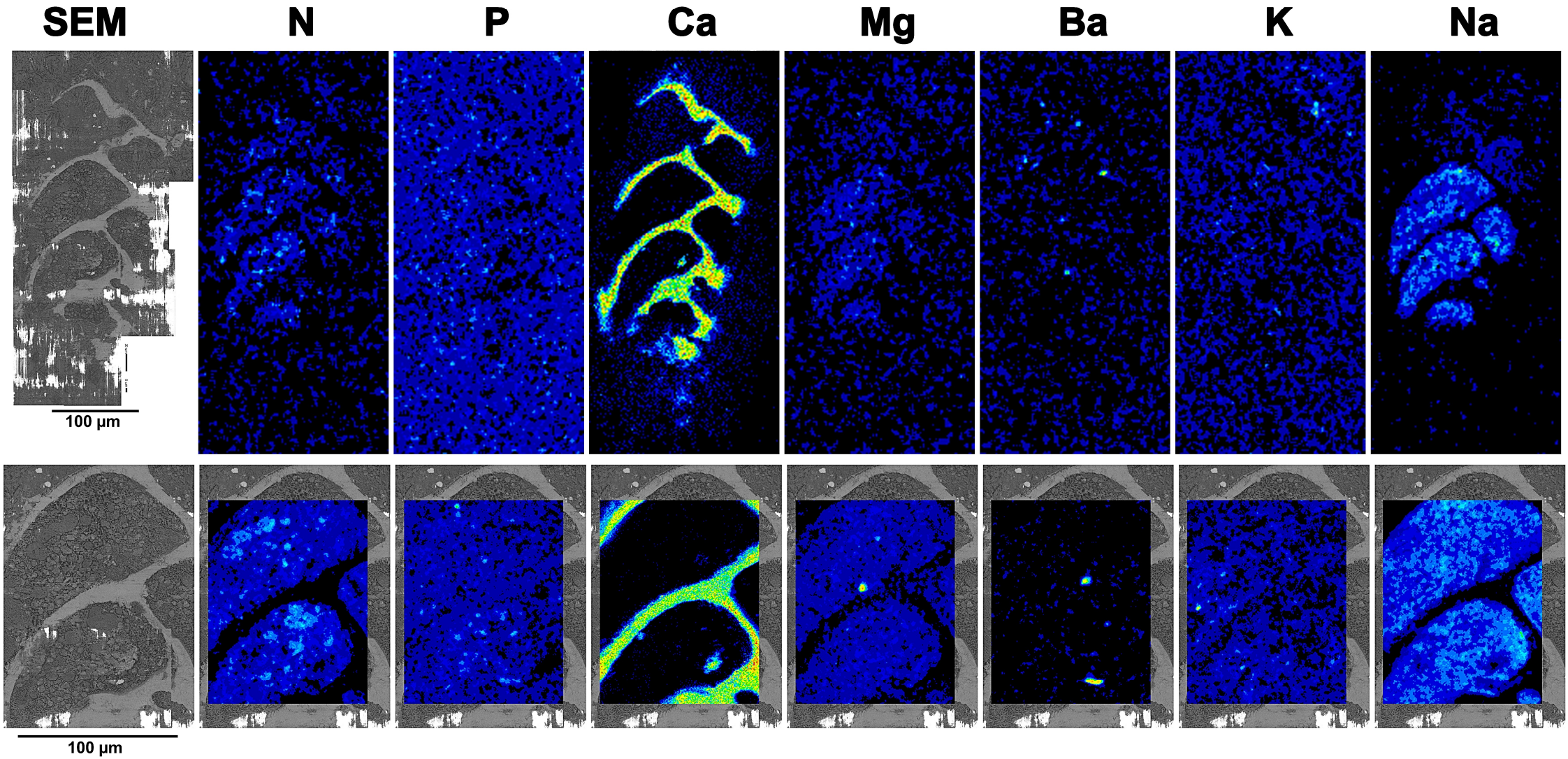

Anoxic0-D

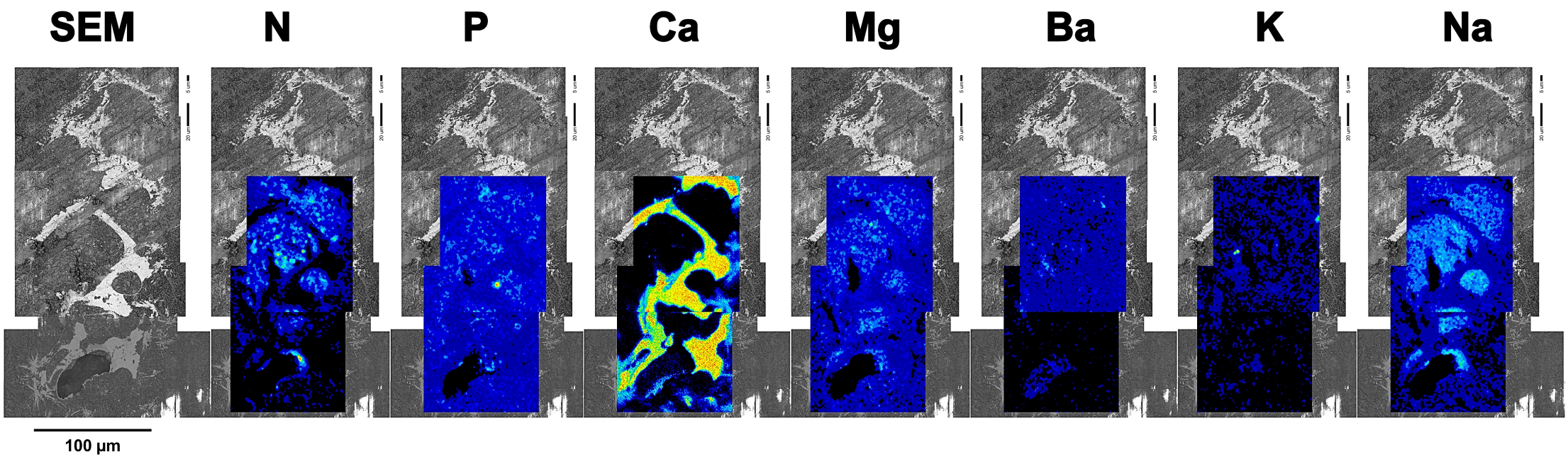

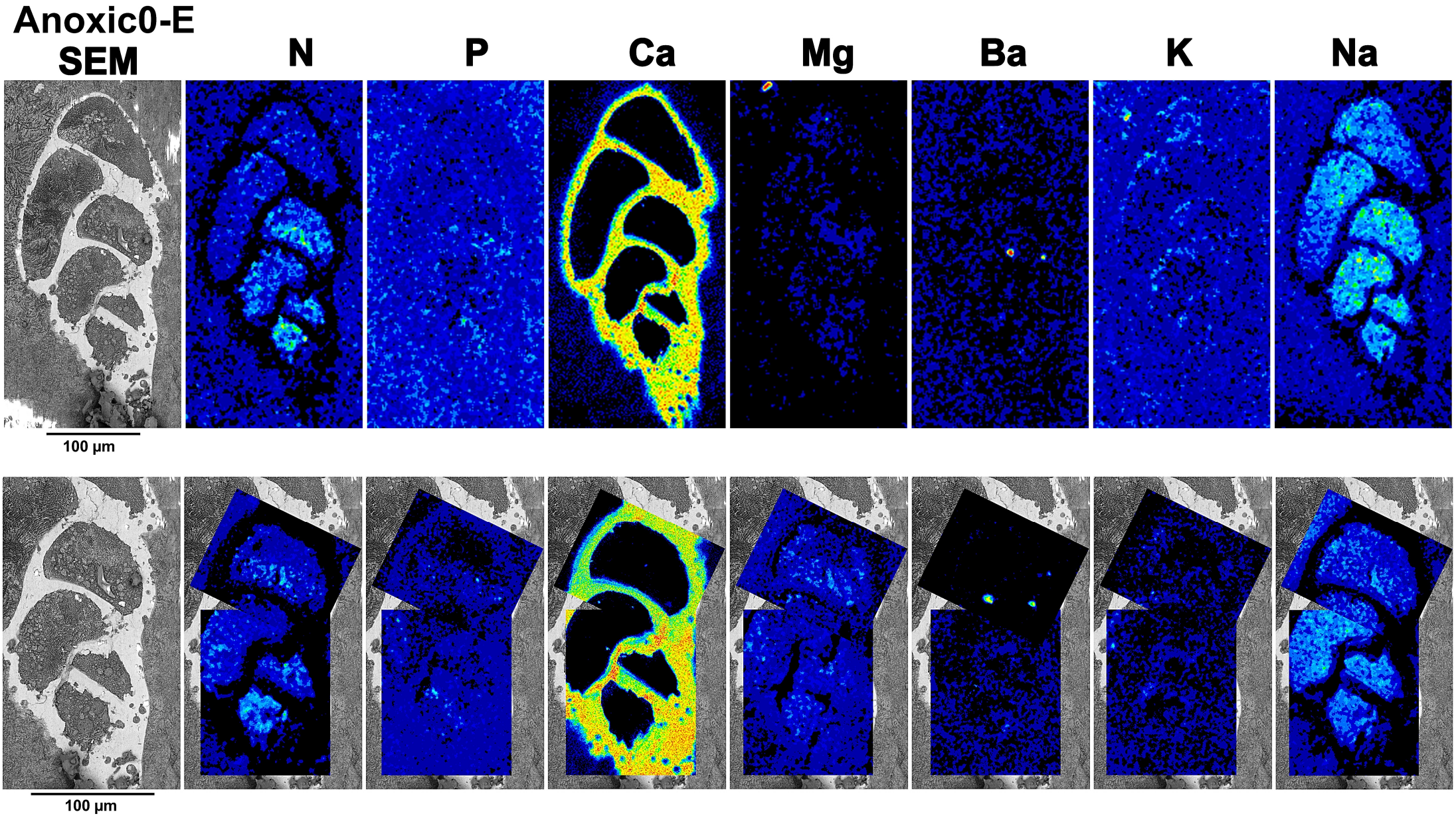
Anoxic0-E
SEM
N
P
Ca
Mg
Ba
K
Na
100 µm
100 µm

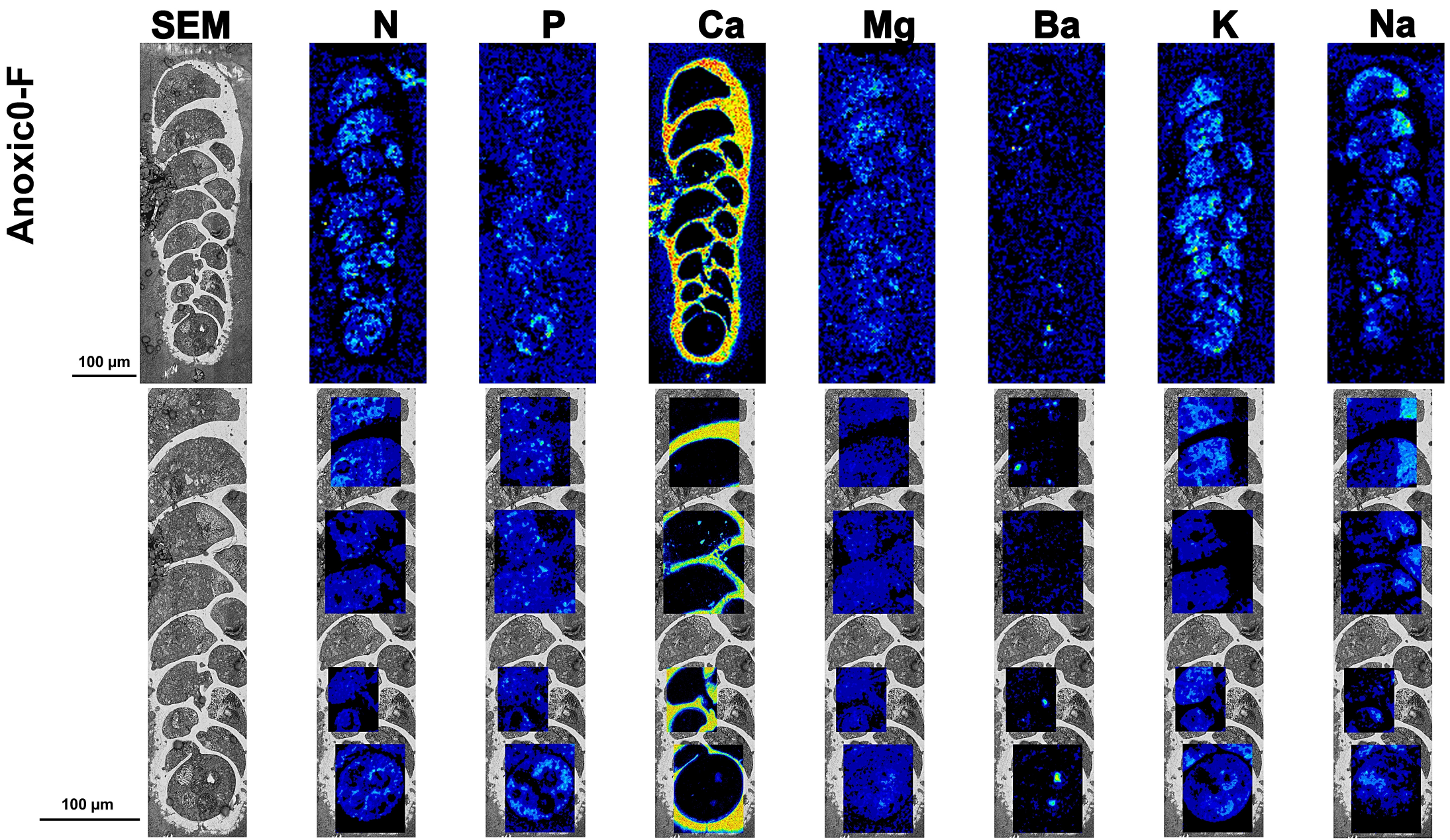
SEM
N
P
Ca
Mg
Ba
K
Na
Anoxic0-F
100 µm
100 µm

Anoxic0-G

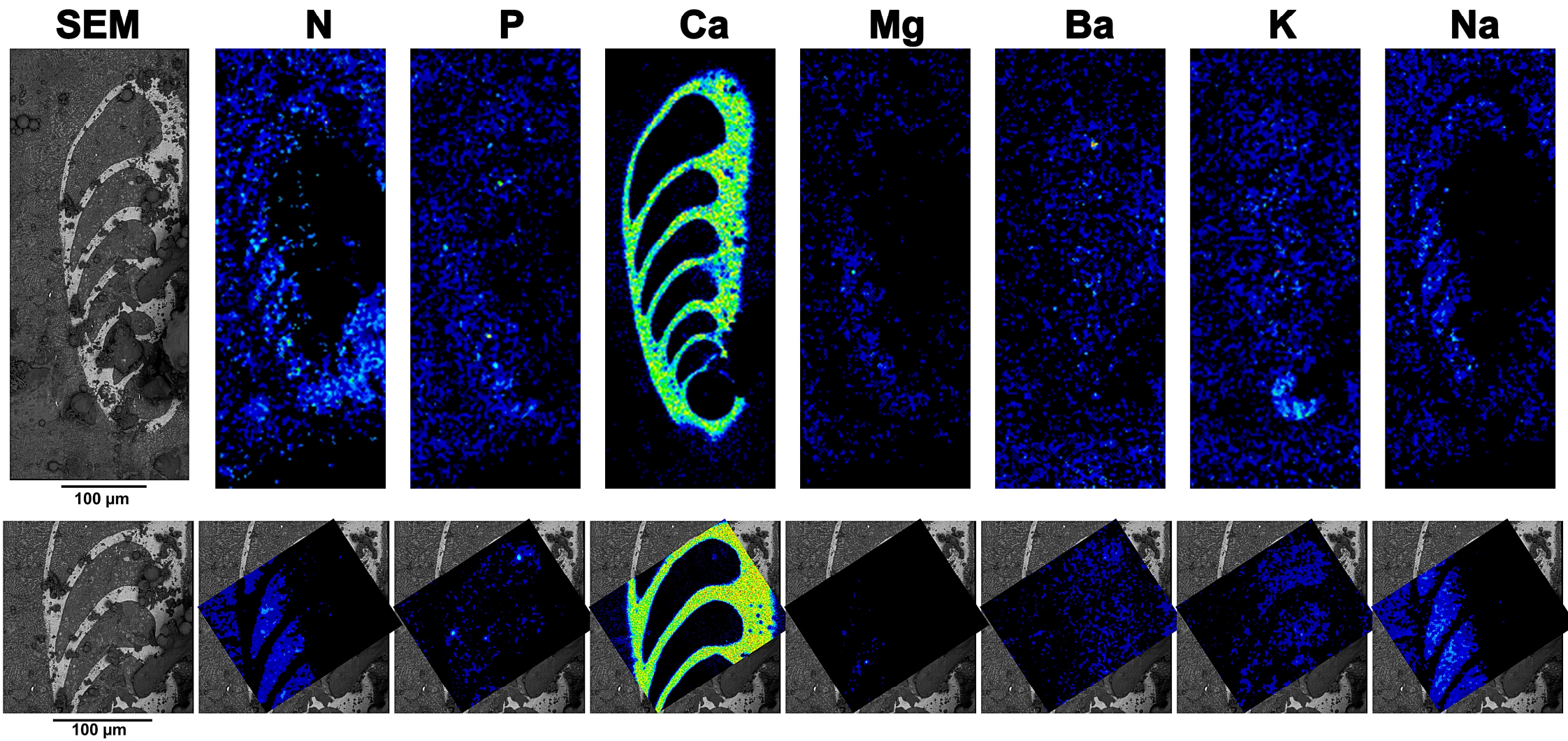

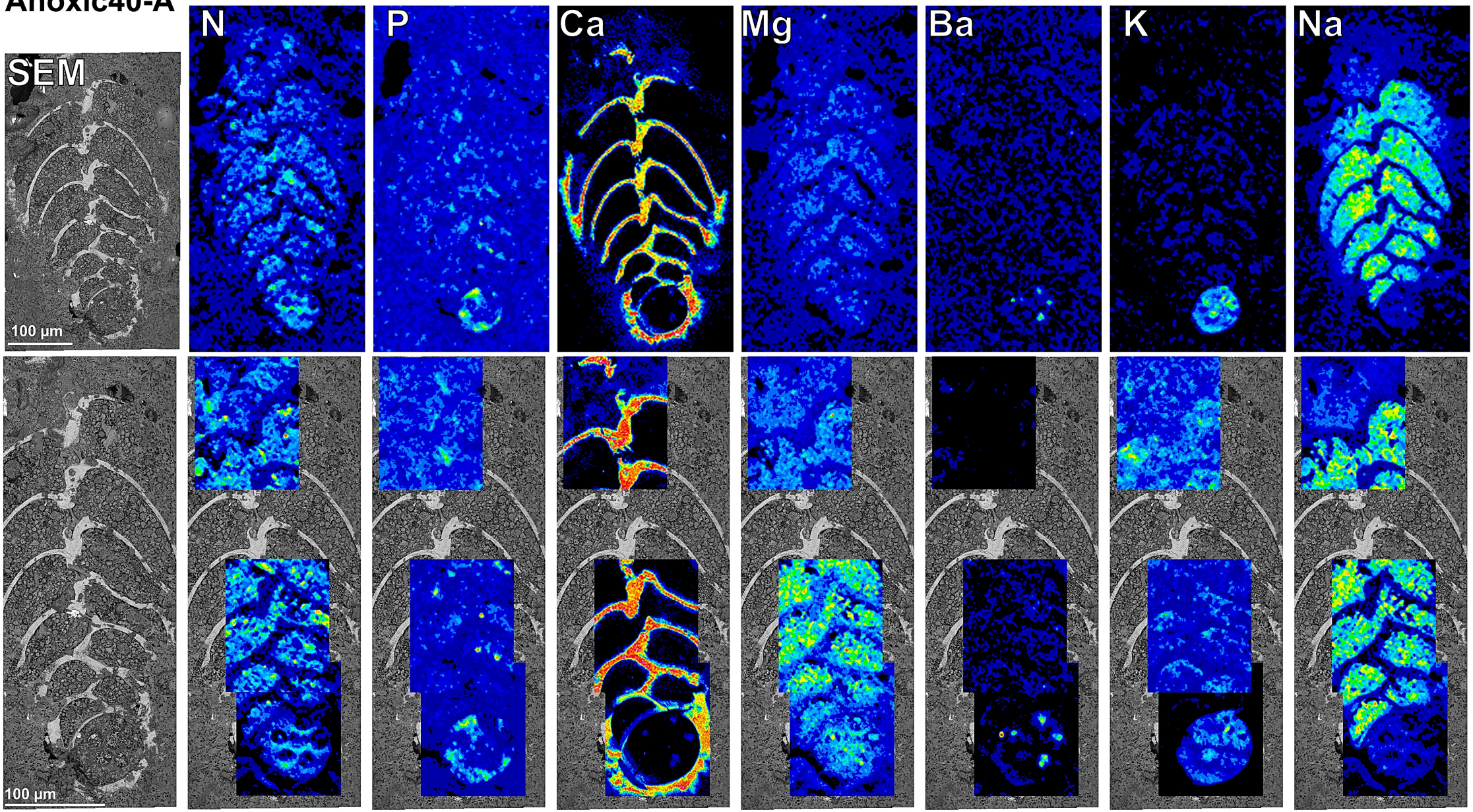
Anoxic40-A
SEM
100 µm
N
P
Ca
Mg
Ba
K
Na
100 µm

Anoxic40-B

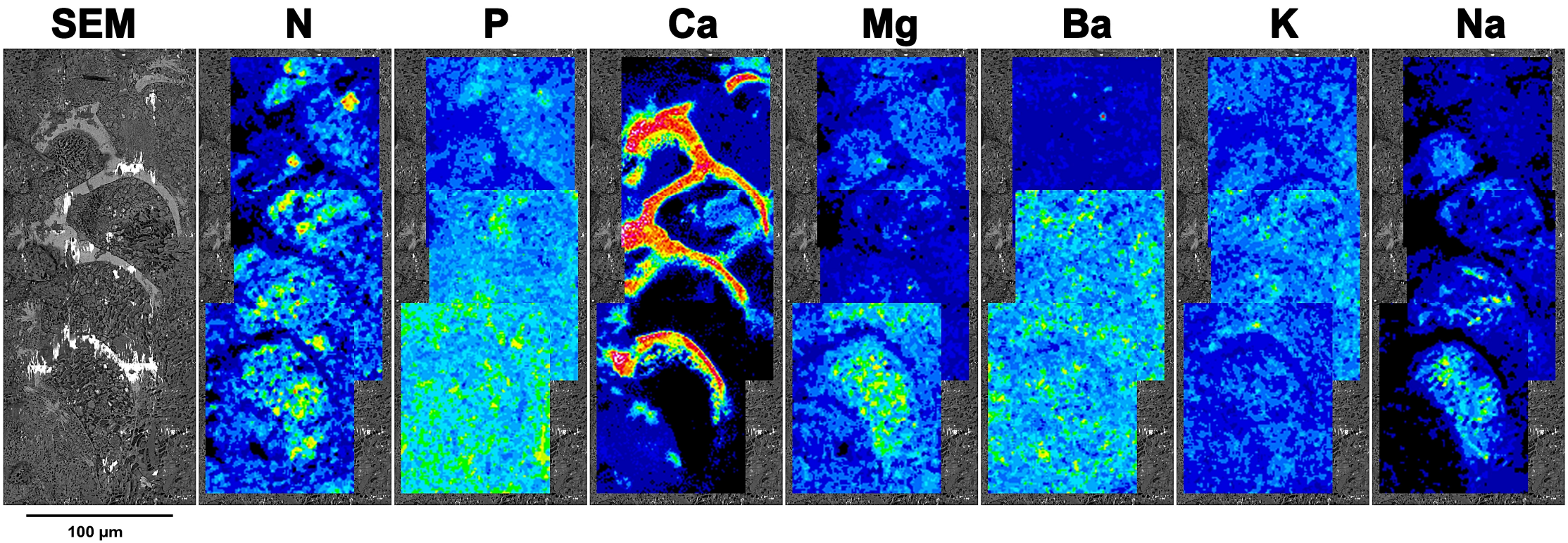

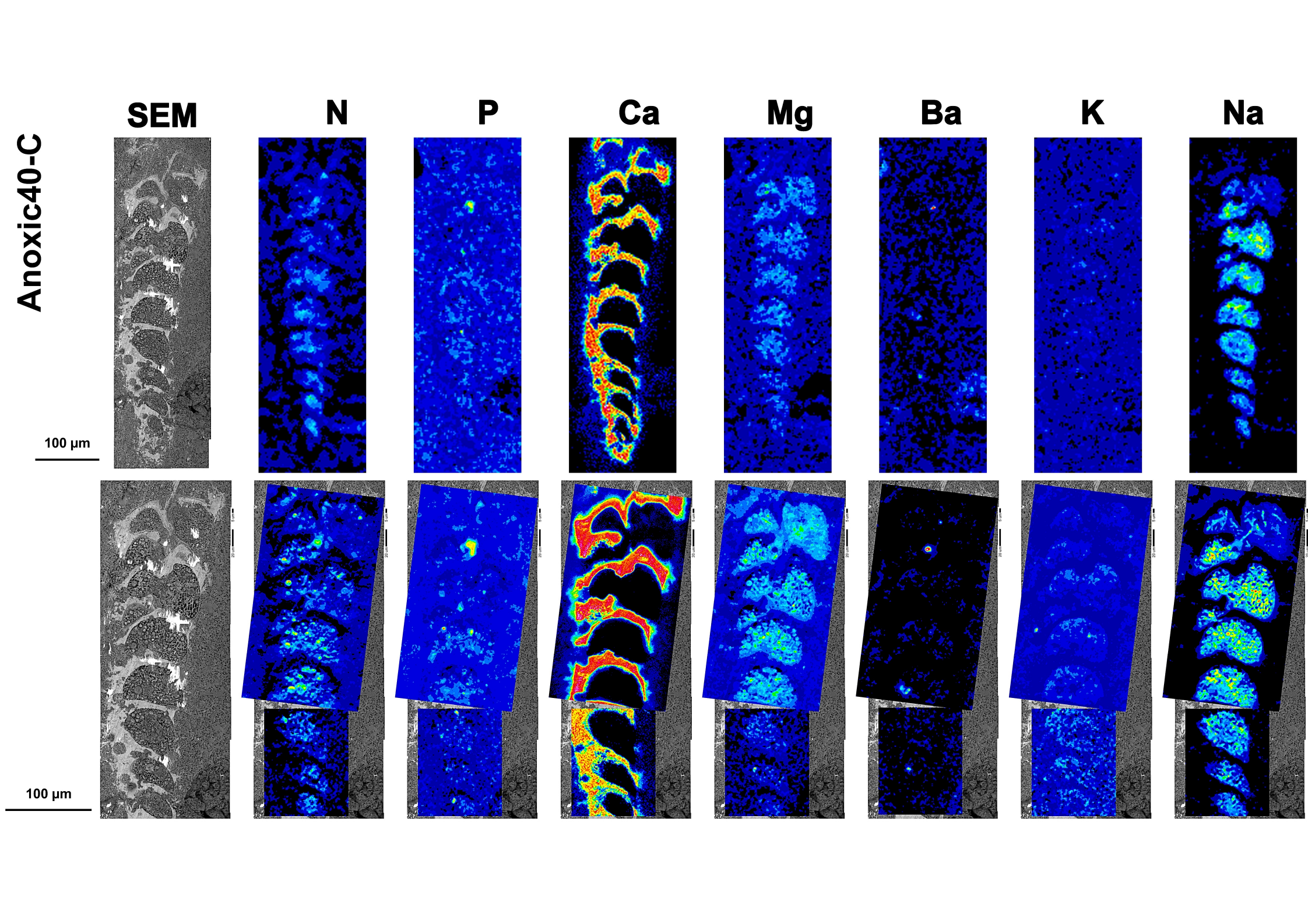
SEM
N
P
Ca
Mg
Ba
K
Na
Anoxic40-C
100 µm
100 µm

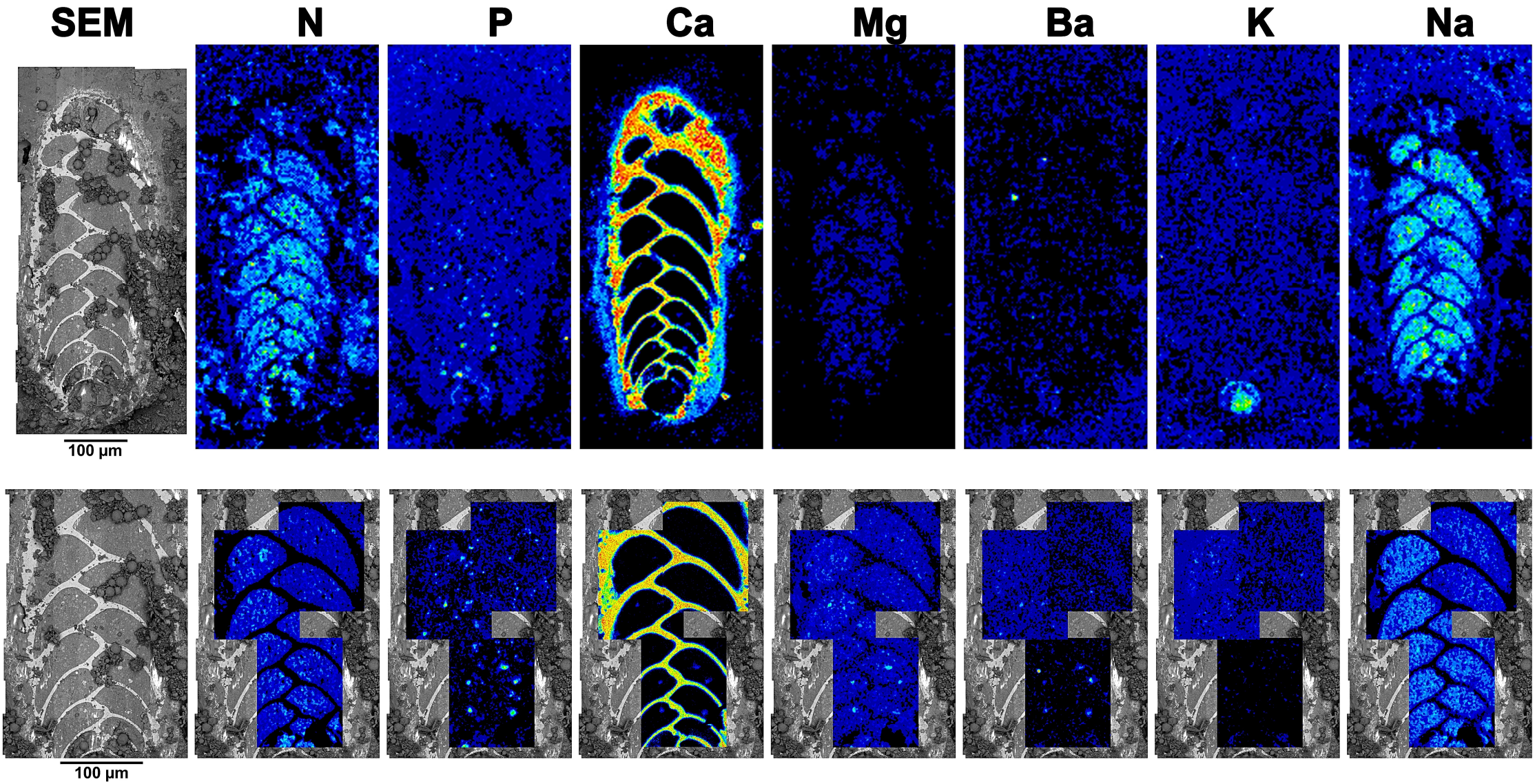
Anoxic40-D
SEM
N
P
Ca
Mg
Ba
K
Na
100 µm
100 µm
